# Developmental and light-entrained expression of melatonin and its relationship to the circadian clock in the sea anemone *Nematostella vectensis*

**DOI:** 10.1186/2041-9139-5-26

**Published:** 2014-08-14

**Authors:** Rafael Peres, Adam M Reitzel, Yale Passamaneck, Solange Castro Afeche, José Cipolla-Neto, Antonio Carlos Marques, Mark Q Martindale

**Affiliations:** 1 Kewalo Marine Laboratory, University of Hawaii, 41 Ahui Street, 96813 Honolulu, HI, USA; 2 Department of Biological Sciences, University of North Carolina at Charlotte, 9201 University City Blvd, Charlotte, 28223-0001 Charlotte, North Carolina, USA; 3 Laboratory of Pharmacology, Butantan Institute, São Paulo, Brazil; 4 Department of Physiology and Biophysics, Institute of Biomedical Sciences, University of São Paulo, Av. Vital Brasil, 1500, Butantã, São Paulo SP, 05503-900, Brazil; 5 Department of Zoology, Biosciences Institute, University of São Paulo. Rua Matao, Trav. 14, 101, 05508-090 Sao Paulo, Brazil; 6 Center for Marine Biology, University of São Paulo. Rodovia Manoel Hypólito do Rego, km. 131,5 - Praia do Cabelo Gordo, São Sebastião SP, 11600-000, CEP, Brazil; 7 Whitney Laboratory for Marine Bioscience, University of Florida, 9505 Ocean Shore Blvd, 32080 St. Augustine, FL, USA

**Keywords:** Circadian clock, Embryogenesis, In situ hybridization, Melatonin

## Abstract

**Background:**

The primary hormone of the vertebrate pineal gland, melatonin, has been identified broadly throughout the eukaryotes. While the role for melatonin in cyclic behavior via interactions with the circadian clock has only been reported in vertebrates, comparative research has shown that the transcription-translation loops of the animal circadian clock likely date to the cnidarian-bilaterian ancestor, leaving open significant questions about the evolutionary origin of melatonin signaling in circadian behavior by interacting with the molecular clock.

**Results:**

Expression of melatonin in adult anemones showed peak expression at the end of light period (zeitgeber time (ZT) = 12) when cultured under diel conditions, coinciding with expression of genes and enzyme activity for members of the melatonin synthesis pathway (tryptophan hydroxylase and hydroxyindol-O-methyltransferase), which also showed rhythmic expression. During embryogenesis and juvenile stages, melatonin showed cyclic oscillations in concentration, peaking in midday. Spatial (in situ hybridization) and quantitative (real-time PCR) transcription of clock genes during development of *N. vectensis* showed these ‘clock’ genes are expressed early in the development, prior to rhythmic oscillations, suggesting functions independent of a function in the circadian clock. Finally, time-course studies revealed that animals transferred from diel conditions to constant darkness lose circadian expression for most of the clock genes within 4 days, which can be reset by melatonin supplementation.

**Conclusions:**

Our results support an ancient role for melatonin in the circadian behavior of animals by showing cyclic expression of this hormone under diel conditions, light-dependent oscillations in genes in the melatonin synthesis pathway, and the function of melatonin in initiating expression of circadian clock genes in the cnidarian *N. vectensis*. The differences in expression melatonin and the circadian clock gene network in the adult stage when compared with developmental stages of *N. vectensis* suggests new research directions to characterize stage-specific mechanisms of circadian clock function in animals.

## Background

Most organisms display daily responses to the periodic changes in the environment, such as light, that include anticipation of these environmental shifts and synchronization of molecular and cellular processes. The diel oscillations in behavior and physiology of animals in response to environmental stimuli are regulated by a combination of hormone expression and molecular networks composing a circadian clock. The contributions of and interactions between each of these two types of mechanisms in circadian behavior and physiology represents a nexus for understanding how different processes regulate organism-environment interactions and how these components may change over evolutionary time.

The primary hormone of the vertebrate pineal gland, the indole melatonin, was first described as a circadian and seasonal output signal of the pacemaker that allows the animal to predict and prepare for changes in their environment [1]. However, many studies have recently been published that expand the importance of melatonin in mammalian and non-mammalian species, including new sites of production, such as the retina [2] and gastrointestinal system [3].

The presence of melatonin in invertebrates was first reported in insects [4], then subsequently in many other invertebrates (see below), and later studies showed the presence of melatonin in a diverse array of eukaryotic organisms, including dinoflagellates [5], in which it may regulate cyst formation [6]; plants, in which it regulates flowering [7]; and fungi [8], where no functions for melatonin have been described so far.

In vertebrates, the peak of melatonin production characteristically occurs during the night [9], and its production is actively regulated by changes in daylight length [1]. However, different patterns of peak production with respect to time of day have been reported for invertebrates. Some protostome species, like flatworms [10] and the fruit fly *Drosophila melanogaster*[11,12]*,* show nocturnal peaks of melatonin, similar to vertebrates. However, in other species, such as the cricket *Gryllus bimaculatus,* melatonin peaks vary depending on the tissue examined, with a nocturnal peak in the brain and in the eyes, but a diurnal peak in the antennae, locomotor appendices and reproductive organs [13]. A similar pattern of differential expression was observed in the gastropod *Aplysia californica*, with a nocturnal melatonin peak in the brain and diurnal peak in the eye [14]. The variation in peak melatonin expression in different organs may be explained in part by melatonin’s role in photo-protection [6].

The molecular pathway that leads to the synthesis of melatonin has been well-characterized in vertebrates. The first step in melatonin synthesis involves the conversion of tryptophan into 5-hydroxytryptophan (5HTP) by the enzyme tryptophan hydroxylase (TPH). The next step involves 5HTP decarboxylation by a nonspecific decarboxylase that transforms it into serotonin. Serotonin is N-acetylated by arylalkylamine N-acetyltransferase (AANAT) producing N-acetylserotonin (NAS) [15]. In rats, AANAT is the rate-limiting enzyme during the daytime, showing a 100% increase in activity during the night. AANAT activity is precisely controlled and decreases sharply, with a half-life of 3 minutes, when sympathetic stimulation is ceased. This response is determined by either the administration of adrenergic antagonists or as a result of nocturnal photostimulation [16-19]. While AANAT-like sequences have been identified in various animal and non-animal lineages [20], the vertebrate type AANAT that functions in melatonin synthesis is restricted to vertebrates following neofunctionalization after duplication of the non-vertebrate AANAT gene [21]. The final step in melatonin production is transformation of NAS to melatonin by hydroxyindol-O-methyltransferase (HIOMT) [22].

In the adult stage of various vertebrate species, one of the main functions of melatonin is as a hormonal output of molecular signaling from an internal clock located in the suprachiasmatic nucleus (SCN) in the brain. The SCN clock is maintained by the oscillations of a set of clock genes (*clock, bmal, cry1*, *cry 2,* and *period*, among others) that controls melatonin production through signaling to the pineal gland [23,24]. Circulating melatonin released by the pineal gland then signals shifts in photoperiod to peripheral clocks in the rest of the body. Also, melatonin acts as a clock input, regulating circadian rhythmicity through the suprachiasmatic nucleus, resetting the clock genes expression [25]. Thus, in vertebrates, melatonin and the circadian clock are tightly associated with maintaining periodic behavior over 24-hour periods.

Although diel oscillations in melatonin production are known from many protostomes, particularly insects (see above), a potential connection between melatonin and the molecular mechanism of the circadian clock is unknown. Comparative research with insects has shown that the circadian clock is composed of similar, and in many cases orthologous, genes that are organized in transcription-translation feedback loops as seen in vertebrates [26-28]. The similarity of the circadian clock mechanism and melatonin synthesis peaks in insects suggests a plausible hypothesis where, as in vertebrates, melatonin and circadian clocks are closely associated.

The ontogeny of melatonin production and circadian clock-related genes and their relationship to circadian rhythms in pre-adult developmental stages remains little studied. Melatonin was recently identified in the eggs of rainbow trout with clear circadian rhythm under a 12 light (L):12 dark (D) photocycle [29], and in bird egg yolk [30]. The presence and potential functions of melatonin during development of invertebrates has not been characterized. Most studies of the developmental expression of genes central to the circadian clock have been conducted in vertebrate species. For example, previous research showed a light-independent initiation of zygotic *per1* transcription on the first day of development of zebrafish [31]. This study also demonstrated that during the first 3 days of development, *clock1* and *bmal1* transcription was not rhythmic, in contrast to later developmental stages where pronounced rhythmic expression in response to light cues was observed. However, direct manipulations show that CLOCK is already functional by regulating transcription of *per1* 24-hours post-fertilization. The differences in embryos and adults suggest a key regulation of the circadian clock in zebrafish development is a product of post-transcriptional processes. Preimplantation mouse embryos express many of the core circadian clock (for example, *bmal, clock, period,* and *cryptochromes*), but expression of these genes did not show 24-hour oscillations in one- to four-cell and blastocyst-stage embryos [32]. The authors of the study hypothesized that circadian genes are not involved in circadian clock regulation in mouse preimplantation embryos but are instead involved in regulation of the cell cycle, particularly meiosis. In *Xenopus* embryogenesis, like mouse, transcripts of the core circadian clock (*bmal1, per 1 and 2,* and *clock),* have expressed in early development, with no evidence of a 24-hour expression cycle, but may be involved in various developmental patterning processes including the formation and timing of somites, heart, and nervous system [33]. These results are consistent with results from *Xenopus* that showed *Clock* expression in the mesodermal cells of the dorsal blastopore lip and the overlying ectodermal cells [34]. At present, there have not been any studies of the ontogeny of melatonin production or clock gene expression in invertebrates.

Together, research in bilaterians has suggested that melatonin is widely present and the molecular mechanisms composing the circadian clock are broadly conserved, but at least in the vertebrates where it has been studied, the interaction of melatonin and the circadian clock in regulating daily behavior is likely restricted to the adult stage. Recent research in cnidarians has begun to identify similar conservation of melatonin production and the circadian clock mechanism. The cnidarians are a phylum of predominately marine species that are the likely sister group to the superphylum Bilateria, which comprises deuterostomes (for example, vertebrates and echinoderms) and protostomes (for example, arthropods and molluscs). Studies utilizing cnidarians as experimental models are therefore useful for determining the antiquity of molecular functions for shared proteins and hormones, as well as for identifying potentially novel features for homologous molecules when compared to bilaterians. Studies of several anthozoan cnidarians has revealed that most of the core circadian clock genes are conserved in cnidarians, and they are differentially expressed in diel lighting conditions [35,36], Despite these similarities, no research to date has addressed whether other mechanisms of the circadian clock may be conserved in cnidarians, particularly the role of hormones in signaling and resetting of the clock. This information is of importance in the understanding of the origin and evolution of melatonin/circadian cycles.

Melatonin has previously been identified in the colonial anthozoan *Renilla köllikeri *[37]*,* where it was concentrated in neuronal cells near gametogenic tissues. Tissue concentration followed a seasonal but not diurnal pattern, suggesting it could serve as a pacemaker involved in seasonal reproduction but not circadian rhythms [8,14]. Data in two other anthozoan species (*Actinia equina* and *Nematostella vectensis*) [38,39] have similarly shown that melatonin is localized near or in gametogenic tissues. Expression patterns of HIOMT and putative melatonin receptors transcripts also showed localized expression in gametogenic tissue as well as surrounding endodermal tissue. In addition, Roopin and Levy [38] showed that melatonin levels in *A. equina* vary on a diel cycle with peak concentration in early subjective night, but the oscillating expression dissipates upon exposure to constant darkness. These later results suggest that melatonin may serve a role in the circadian clock because cnidarians lose oscillations in the expression of circadian clock genes when removed from an entraining light cue [35,36]. Together, these previous data from cnidarians suggest an unresolved picture for potential role(s) of melatonin in cnidarians, which may include both seasonal and daily functions. Melatonin in cnidarians has not been studied in pre-adult stages to determine if it is produced in stages lacking gametogenic tissue and, if expressed, whether concentration varies in a pattern consistent with a role in circadian behavior.

The present study analyzes the presence of melatonin and its rhythmic oscillation in adults cultured under different lighting regimes as well as developmental stages of the starlet sea anemone *Nematostella vectensis*. We also report the expression of genes encoding the enzymes of the melatonin pathway described from vertebrate species, extending previous results for *N. vectensis* and *A. equina *[38,39]*.* We also studied the spatial (in situ hybridization) and quantitative (real-time PCR) transcription of clock genes in the development of *N. vectensis*, showing that the clock genes begin to be expressed early in the development, prior to rhythmic oscillations, suggesting functions independent of a function in the circadian clock. Finally, we experimentally determine the impact of melatonin supplementation on the transcription of circadian clock genes in this cnidarian, which strongly suggests that the function of melatonin in resetting of the circadian clock is conserved by resetting transcriptional oscillations that dissipate after a few days of constant darkness. Together, our data support hypotheses that the function of melatonin in the circadian clock dates back to at least the cnidarian-bilaterian ancestor, the genes that compose the circadian clock have developmental functions in embryogenesis, and larval *N. vectensis* potentially have diel rhythms evidenced by temporal expression by melatonin.

## Methods

### Animal culturing and experimental design

Adult *N. vectensis* (measuring between 2 and 5 cm in length) were kept under a 12-hour:12-hour light–dark cycle (lights on at 7:00 h (Hawaii standard time); Zeitgeber Time ZT = 0; lights off at 19:00 h; Zeitgeber Time ZT = 12 - under full spectrum lights, (Corallife 50/50 bulb, approximately 3,600 lux), in glass bowls with 13 parts per thousand (‰) filtered local seawater (diluted with distilled water, referred to as ‘1/3X seawater’) in a temperature-controlled incubator (17°C). Water was changed every day (ZT = 0), and animals were fed twice weekly with freshly hatched brine shrimp. A second group of adult individuals were kept in constant darkness for 20 days under the same conditions, with water changes also occurring at ZT = 0. For a second experiment, to measure dissipation of transcriptional oscillations in animals removed from light cycling, groups of animals were kept in constant darkness for 24 h, 48 h and 96 h and otherwise with the same conditions.

For the melatonin treatments, adult animals were kept in constant darkness for 5 days and then exposed to 0.1 μM melatonin (Sigma Chemical Co., St. Louis, MO, USA) for the subsequent 15 consecutive days in constant darkness. Exogenous melatonin addition was administered at the time of the transition to the subjective night (ZT = 12), corresponding to a time of peak melatonin production (see Results). After 12 hours of incubation, the animals were rinsed in fresh 1/3X seawater and placed in new dishes for the remainder of the experiment.

To determine the temporal production of melatonin in adults, embryos (48-hour post-fertilization), and juveniles (1-week and 2-week post-fertilization), animals were individually collected from their respective treatment every 3 hours and placed in 1.5 ml microcentrifuge tubes and flash frozen with liquid nitrogen. Embryos and juveniles were also kept from egg stage under the same condition of the adults in 12-hour:12-hour light–dark cycle. Eggs were fertilized in the ZT = 6. Individuals for RNA extractions were individually placed into a 1.5 ml tube with TriPure Reagent (Roche Inc, USA), then flash frozen.

### In situ hybridizations

In situ hybridizations were performed as previously described [40]. Embryos were collected from specific time points post-fertilization (24, 48, 96, 144 and 168 hours - polyps were not fed during the stages). Eggs were fertilized in the ZT = 6. The animal collection was also at ZT = 6, occurring 24, 48, 96, 144 or 168 hours after fertilization and corresponding with the developmental stages - blastula, late blastula, gastrula, planula, and late planula, respectively. All stages were fixed in fresh ice-cold 3.7% formaldehyde with 0.2% glutaraldehyde in 1/3X seawater for 60 seconds and then postfixed in 3.7% formaldehyde in 1/3X seawater at 4°C for 1 hour. Fixed embryos were rinsed five times in PBS buffer plus 0.1% Tween 20 (PTw) and once in deionized water, and transferred to 100% methanol for storage at -20°C. Early embryos were removed from the jelly of the egg mass by treating with freshly made 2% cysteine in 1/3X seawater (pH 7.4 to 7.6) for 10 minutes. Planula and late planula were relaxed in 7% MgCl2 in 1/3X seawater for 10 minutes prior to fixation. In situ hybridization using 1 to 2 kb digoxigenin-labeled riboprobes for *Clock*, *Timeout*, and *Cryptochromes* were performed to determine the spatial and temporal distribution of transcripts in each stage. Probe concentration ranged from 0.5 to 1.0 ng ml^-1^, and hybridizations were performed at 70°C for 40 hours in 50% formamide. Probe detection was achieved by incubation with an antidigoxigenin antibody conjugated to alkaline phosphatase (Roche, Inc). Subsequently, the presence of alkaline phosphatase was detected by a colorimetric detection reaction using the substrate NBT-BCIP. Specimens were photographed on a Axioskop II with a Zeiss Zxiocam HRc.

#### ***Melatonin assays***

Levels of melatonin (nanograms) were assayed in *N. vectensis* (adults and embryos) by a modified and highly sensitive enzyme-linked immunosorbent assay (ELISA) method (IBL International, Hamburg, Germany). Melatonin was extracted from tissue with 0.6 ml of 0.6% perchloric acid. To normalize the assays, 2 μl of each sample were used for protein measurements. The amount of total protein (in milligrams) was determined spectrophotometrically at 280 nm with a NanoDrop 1000 (Thermo Scientific, Inc.). Samples were then passed through a C18 reversed-phase column, extracted with methanol, evaporated to dryness, and reconstituted with water. Each sample was added to a microplate well coated with the goat-anti-rabbit anti-melatonin antibody. An unknown amount of antigen present in the sample and a fixed amount of enzyme-labeled antigen competed for the binding sites of the antibodies coated onto the wells. After incubation for two hours, the wells were washed to stop the competition reaction. The substrate p-nitrophenyl phosphate (PNPP) was added and the concentration of antigen determined as the inverse proportion of the optical density measured in a photometer. Melatonin standards were used to construct a standard curve against which the unknown samples were calculated. Specificity of the method is close to 100% where melatonin concentrations exceed 3 pg ml-1, the limit of sensitivity for this assay. (IBL ELISA kit manual). A previous test adding known concentrations of melatonin to the *N. vectensis* samples showed the recovery from our isolation protocol was approximately 85% (data not shown).

For additional validation, the presence of *bona fide* melatonin was confirmed by high performance liquid chromatography (HPLC) with electrochemical detection (Chromeleon™ 7.1, Dionex System, Sunnyvale, CA, USA). Melatonin was separated on Acclaim C18 column (2,2 μM 2.1 × 100 mm, Dionex System, Sunnyvale, CA, USA). The chromatographic system was isocratically operated (that is, system set to not change composition of the solution during the run) with the following mobile phase: 0.1 M sodium acetate, 0.1 M citric acid, 0.15 mM EDTA, 32% methanol, pH 3.7, at a 0.120 mlmin^-1^ flow rate. The electrochemical detector potential was adjusted to +750 mV. The elution time for melatonin was approximately 10 minutes. Each tissue sample was sonicated (Microson XL 2005, Heat System Inc., Farmingdale, NY, USA) in a solution of 0.1 M perchloric acid (200 μl) containing 0.02% EDTA and 0.02% sodium bisulfate. After centrifugation (2 min, 13,000 g*,* Eppendorf 5415C Centrifuge, Brinkman Instruments Inc., Westbury, NY, USA), 40 μl of the supernatant was injected into the chromatographic system with an automatic injector. A melatonin stock solution was prepared in 0.1 M HCL with 0.02% EDTA and 0.02% sodium metabisulfite, and then dilutions to make a standard curve (0.145 to 4.64 ng 20 μl^-1^) were prepared with perchloric acid just before the assays were performed. Blank assays containing no melatonin were used as negative controls.

### Serotonin assays

Serotonin (5HT) content was quantified using a sensitive enzyme-linked immunosorbent assay method (IBL International, Hamburg, Germany). The embryos samples (approximately 60 embryos per sample) were centrifuged (3,000 × g for 2 min) and the sea water removed. Samples were reconstituted in 120 μl of the assay buffer of the kit. To normalize the assays, 2 μl of each sample was used to protein measures. Briefly, the assay was performed as follows: 20 μl of samples and 5HT standard dilutions were applied to 96-well microtiter plates previously coated with goat anti-rabbit antibody followed by 50 μl biotin-labeled 5HT and 50 μl rabbit antibody against 5HT. After incubation overnight at 4°C and washing with wash buffer, 150 μl of fresh prepared enzyme conjugated was added. Samples from the developmental stages, standards, positive and negative controls were incubated for 1 hour at room temperature with gentle mixing, washed again, then 200 μl p-nitrophenylphosphate substrate was added, and the enzyme reaction was terminated after 60 minutes by addition of 50 μl of p-nitrophenylphosphate stop solution. Absorption was measured at 405 nm in a spectrophotometer and the concentration of 5HT was calculated from the reference curve. A previous test adding known concentrations of serotonin to the *N. vectensis* samples showed us that the recovery was approximately 90% (data not shown).

### Total RNA extraction, reverse-transcription reaction, and PCR

Total RNA was extracted from samples using TriPure Reagent (Roche Inc, USA) according to the manufacturer's specifications. Briefly, adult animals/embryos were lysed in 0.5 ml TriPure reagent and incubated for 5 min at room temperature. Two-hundred μl of 1-bromo-3-chloropropane (BCP) was added to the tubes and centrifuged at 12,000 × g for 15 minutes. The aqueous phase was transferred to a fresh tube, treated with DNAse (Ambion, Life Technologies, Grand Island, NY, USA) and total RNA was pelleted by precipitation with isopropyl alcohol and centrifugation (12,000 × g for 10 min). The RNA pellet was washed with ethanol (75%) and pelleted at 7,500 × g for 5 min and air-dried at room temperature. RNA pellets were reconstituted in RNase-free water. RNA was quantified spectrophotometrically at 260 nm with 260/280 ratios between 1.8 and 2.0. RNA quality was also checked by 1.4% agarose gel electrophoresis stained with 5 μg ml^-1^ ethidium bromide. Complementary DNA (cDNA) was synthesized with the Advantage RT-for-PCR Kit protocol (Clontech Laboratories, Mountain View, CA, USA) following the supplier's instructions. cDNA was stored in water at -20°C.

Primers for quantitative PCR were designed for genes of interest to amplify gene fragments of length 75 to 150 bp [see Additional file 1] using MacVector (MacVector, Inc, North Carolina USA). The genes studied include presumptive circadian clock genes previously reported by Reitzel et al. [35], as well as identified genes likely involved in the melatonin synthesis pathway [39]. Ribosomal protein P0 [XM_001626244.1] was used as a normalization gene for all experiments. Previous qPCR assays showed that this gene does not have variation in transcription, either between different time points within a treatment or between different treatments. The cycling parameters used were 10 seconds at 95°C, 20 seconds at specific annealing temperature for each primer [see Additional file 1], and 20 seconds at 72°C during 45 cycles. qPCR was conducted on a LightCycler™ 480 Real-Time PCR System (Roche, Inc.) using SYBR Green I Master mix (Roche, Inc.).

### Enzyme activity assays

Tryptophan hydroxylase (TPH) activity was quantified as previously described [41]. Briefly, each tissue sample was sonicated in sodium phosphate buffer (2 mM, pH 7, 100 μL), and the following mixture was added to each sample: HEPES (50 mM, pH 7), catalase (100 μg ml^-1^), tryptophan (50 μM), dithiothreitol (5 mM), Fe (NH_4_)^2^ (SO_4_)^2^ (10 μM), 6-MPH4 (500 μM) and 1 μL of [3^H^] tryptophan (1 mCi ml^-1^ - previously dried under nitrogen). The material was incubated at 37°C for 10 minutes. An activated charcoal solution was added (7.5% in 1 M HCl) to terminate the reaction. Two-hundred μL of the supernatant was transferred to scintillation vials, and radioactivity was determined by a Beckman LS6500 β counter (Beckman Coulter Inc., CA, USA). Positive (rat pineal glands) and negative (water) controls were included in each assay.

Hydroxyindol-O-methyltransferase (HIOMT) activity was assayed as previously described [22]. Samples were sonicated in phosphate buffer (0.05 M, pH 7.9, 50 μL). One hundred fifty μL of a solution containing ^14^C-S-adenosyl-L-methionine (specific activity 43.8 mCi - Sigma Chemical Co., St. Louis, MO, USA) and N-acetylserotonin (1 mM) was then added. The homogenates were incubated for 30 minutes at 37°C. The reaction was terminated by adding 200 μL of sodium borate buffer (12.5 mM, pH 10) and 1 mL of buffer saturated chloroform. The tubes were centrifuged at 13,000 x g for 5 minutes at 4°C. The ^14^[C] melatonin product was extracted in 800 μL of chloroform, air-dried, and the radioactivity was determined by a Beckman LS6500 β counter (Beckman Coulter Inc., CA, USA). Positive (rat pineal glands) and negative (water) were included in each assay. To normalize the TPH and HIOMT assays, 2 μl of each sample were used quantify protein concentration to the TPH and to the HIOMT experiments. The results are presented as the ratio of activity per milligram of protein.

#### ***Statistical analysis***

Data are presented as the mean of four independent replicates ± SEM. Melatonin and serotonin content was expressed as ng mg-1 of protein. Statistical analyses (GraphPad Prism 5.0, GraphPad Software Inc., San Diego, CA, USA) were performed using an ANOVA (one- or two-way, as required) followed by Bonferroni post-hoc test.

One-way ANOVA was used to evaluate the influence of the variable ‘time of day’ on each temporal series. Given an overall significant one-way ANOVA, the cosinor method was utilized to evaluate the presence of a daily 24-hour rhythm. The theoretical cosine curve fitting was applied in each temporal series using the least-square calculation. The best fitting curve for each day was determined with F-statistics. The null hypothesis tested was zero amplitude, that is, no rhythmicity at a 24-hour frequency. For each temporal series, three parameters of the adjusted curve were calculated: acrophase (time of the maximum value of the adjusted curve), mesor (value of the mean level of the adjusted curve) and amplitude (distance between the mesor and the maximum or minimum value of the adjusted curve). These rhythmic parameters were compared between groups using the Student’s *t*-test [42]. All statistical analyses were considered significant for *P* ≤0.05.

## Results

### Melatonin concentration in adults

**Figure 1:**
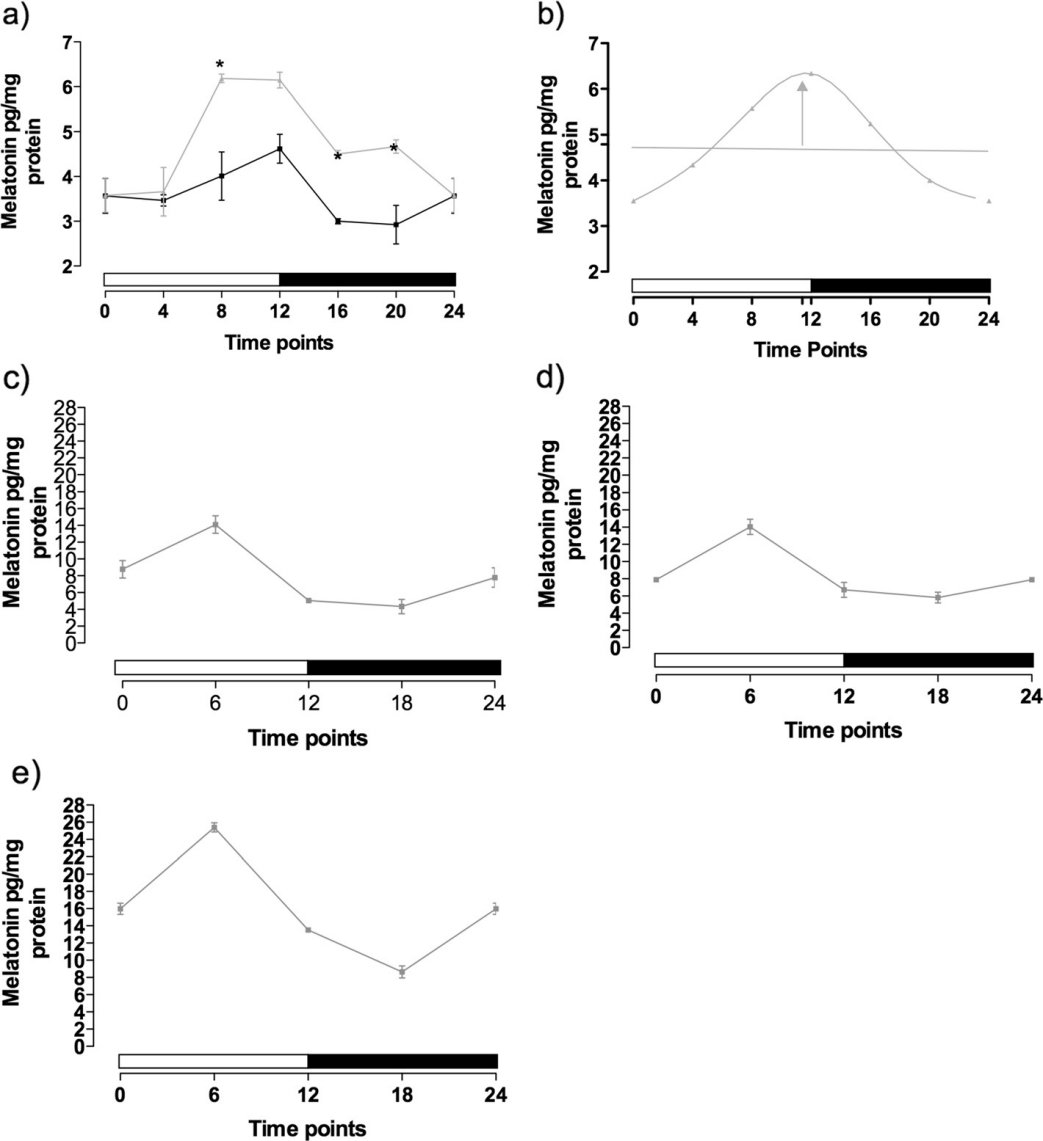
**Melatonin content in *Nematostella vectensis.*** Results in **(a)***Nematostella vectensis* adults cultured in a light–dark (light gray line) (12:12 h) or constant darkness cycle (black line); and in 48-h **(c)**, 1-week- **(d)** and 2-week-old **(e)** embryos in light dark cycle. *P <0.05. Two-way analysis of variance (ANOVA) followed by Bonferroni multiple comparison (n = 5 individuals/samples per time point, per group). **(b)** Curve obtained with the cosinor test with the adult animals cultured in the light–dark cycle. The horizontal line, the mesor, represents the mean oscillation, and the vertical arrow, the acrophase, the time of occurrence of the daily-peak of melatonin.

Melatonin was successfully detected in adults and embryos using the ELISA kit. High performance liquid chromatography (HPLC) with electrochemical detection assay confirmed the presence of melatonin, with the same retention time for the indol in the standards and in all samples [see Additional file 2]. For the adults in the light–dark treatment, we observed peak expression at 4 hours before the transition to the dark phase (that is, ZT = 8, Figure 1a, light-gray line) that was sustained until the ZT = 20 (4 hours before the transition to subjective day phase). In order to evaluate if these oscillations in melatonin production were a response to the presence or absence of light, we repeated the experiment using animals that were kept in constant darkness for 20 days. After 20 days of constant darkness, melatonin still showed significantly higher concentration in the transition from the subjective day to the subjective night (ZT = 12, Figure 1a, black line, one-way ANOVA, *P* = 0.0124). However, the night peak of melatonin was not sustained during the subjective night where we measured significantly lower concentrations of melatonin in ZTs = 16 and 20. This observation is confirmed by rhythmic analysis using the cosinor procedures, which showed that melatonin levels cycled in the light–dark cycle, but not in constant darkness (Figure 1b).

### Melatonin and serotonin concentration during development

For *N. vectensis* embryos, we measured a peak expression of melatonin 6 hours after lights on (ZT = 6) in 48-hour embryos (Figure 1c) as well as juvenile polyp stages (1-week post-fertilization, Figure 1d; 2-weeks post-fertilization, Figure 1e). The peak amount of melatonin was similar in the 48-hour (14.11 ± 1.04 pg mg^-1^ f protein) and 1-week juveniles (14.04 ± 0.88 pg mg^-1^ of protein) but was almost the half of the amount observed in the 2-week juveniles (25.41 ± 0.54 pg mg^-1^ of protein). The lowest melatonin concentration was observed in the middle of the dark period (ZT = 18) for all the groups (4.34 ± 0.86 pg mg^-1^ of protein for the 48-hour; 5.81 ± 0.62 pg mg^-1^ of protein for 1-week and 8.64 ± 0.701 pg mg^-1^ of protein for 2-week juveniles).

**Figure 2:**
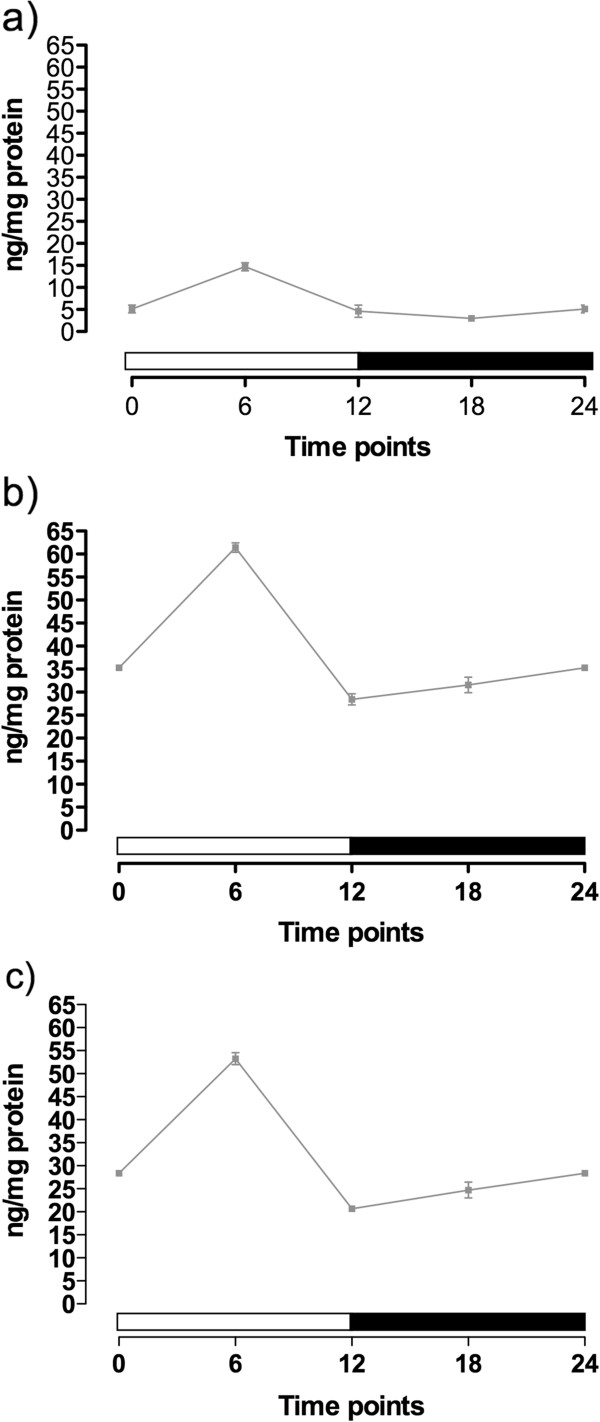
**Serotonin content in *Nematostella vectensis *embryos cultured in a light–dark (12:12 h) cycle.** Samples from 48-h **(a)**, 1-week **(b)** and 2-weeks **(c)** post-fertilization.

To better understand the dynamics of the melatonin production in the embryos of *N. vectensis*, we also made serotonin measures on the same developmental time points. Serotonin was successfully detected in all samples. The pattern of production replicates the one showed in the melatonin assays. Again, there was a peak expression 6 hours after lights on (ZT = 6) in the 48-hour embryos (Figure 2a), 1-week (Figure 2b) and 2-week-old polyps (Figure 2c). The lowest serotonin peak was observed in the 48-hour embryos (14.73 ± 0.91 pg mg^-1^ of protein). In the 1-week juveniles, the peak was 61.43 ± 1.02 pg mg^-1^ of protein, which was higher than the 2-week juveniles (53.25 ± 1.30 pg mg^-1^ of protein). This difference between the juvenile stages potentially indicates a higher amount of serotonin is being converted in melatonin in these older juveniles.

### Diel oscillations in melatonin production pathway

In order to characterize the melatonin production in *N. vectensis*, we identified and measured the expression pattern of transcripts coding for enzymes in the classic pathway of melatonin production*.* By searching the *N. vectensis* gene models and EST library (Joint Genome Institute *Nematostella* genome browser, http://genome.jgi-psf.org/Nemve1/Nemve1.home.html), we identified candidates for the first and the last enzymes in the classical melatonin pathway, tryptophan hydroxylase [XM_001623795.1] and hydroxyindol-O-methyltransferase [XM_001627179.1]. HIOMT has been previously described in *N. vectensis *[39]*.* Both genes were amplified, cloned and sequenced, with the same nucleotide sequence present in the EST library [see Additional file 3 and Additional file 4]. The sequences had high similarity to sequences previously described in other species. Phylogenetic analyses show that the candidate *N. vectensis* TPH gene groups with PaH (phenylalanine hydroxylases), the sister group to TPH, with no clear TPH ortholog in the genome [Additional file 5]. Because the *N. vectensis* TPH-like gene groups with PaH, we refer to it as TPH/PaH, indicating its co-orthologous relationship to both TPH and PaH from bilaterians. *N. vectensis* HIOMT groups with previously reported HIOMT proteins among various animal species. The phylogenetic analysis of the *N. vectensis* HIOMT gene clearly supports orthology with other animal HIOMT genes to the exclusion of non-animal genes (o-methyltransferases). Human and zebrafish HIOMT-like genes are supported as more recent duplication events in the deuterostome or chordate lineage [see Additional file 6].

**Figure 3:**
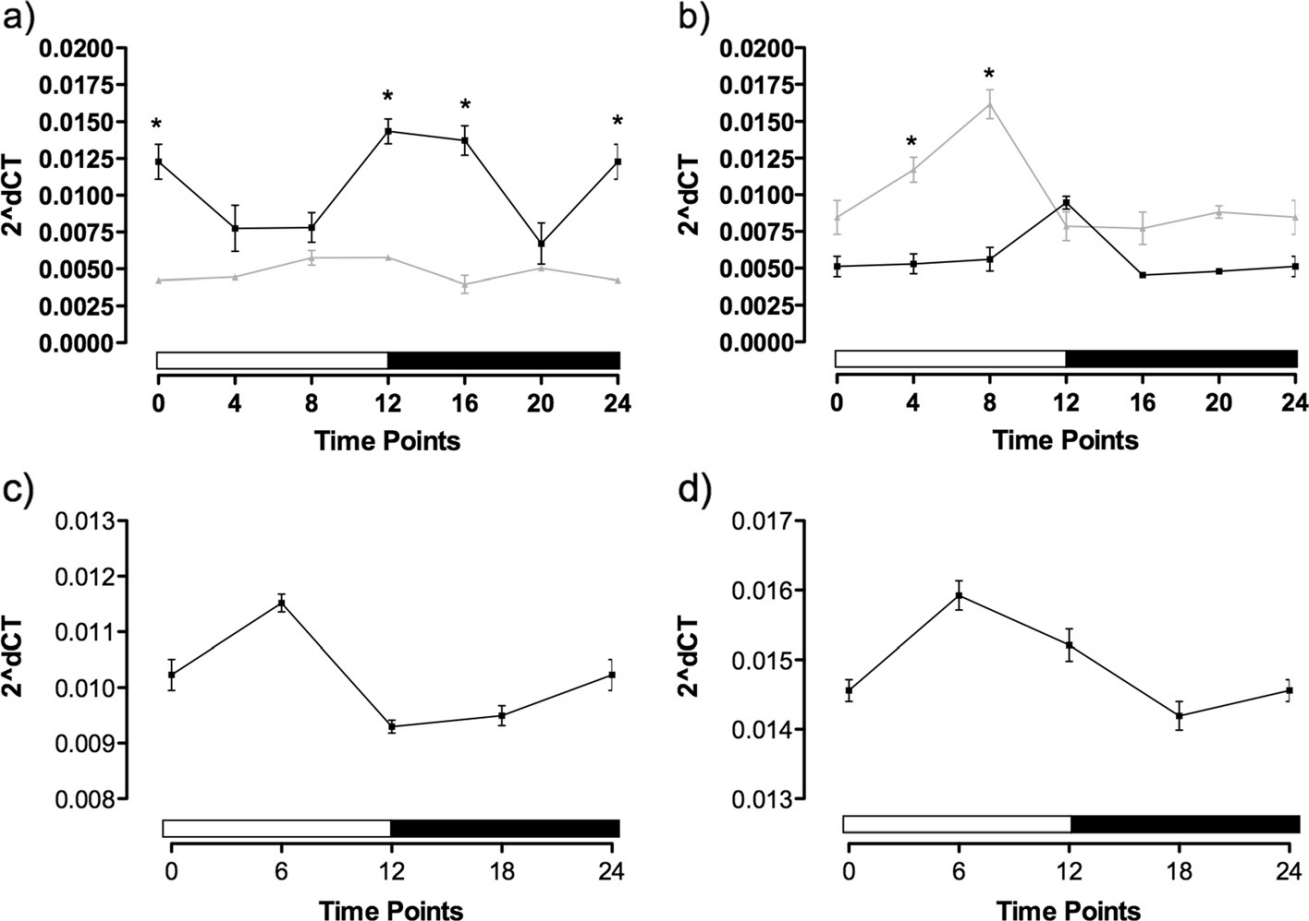
**Relative mRNA expression of melatonin pathway production in *Nematostella *vectensis.** Results of **a)** tryptophan hydroxylase (TPH)/phenylalanine hydroxylases (PaH) and **b)** hydroxyindol-O-methyltransferase (HIOMT) in adult individuals of *Nematostella vectensis* kept in a light–dark (12:12 h) or constant darkness cycle; and **c)** TPH/PaH and **d)** HIOMT expression in 2-week-old embryos. Data were normalized by the gene to the ribosomal protein P0. Each point represents five individuals/samples. *Statistically different from light–dark with *P* <0.05.

NvTPH/PaH showed little change in expression in adult animals exposed to light–dark conditions. There was a moderate, but measurable, increase in transcription before the transition from the light to the dark period (Figure 3a, light-gray line), which correlates with the pattern observed in the melatonin production. There was significant variation in expression of TPH/PaH in the animals in constant darkness in a pattern with two peaks that does not resemble the one observed for melatonin (Figure 3a, black line). The Pearson correlation test between the TPH/PaH expression and melatonin content was positive for the animals in the light–dark cycle (r = 0.871, *P* = 0.0053). There was no correlation for the animals in constant darkness (r value = 0.286, *P* = 0.267). Expression of TPH/PaH in the 2-week embryos showed a pattern that again correlated with the serotonin and melatonin production profile (Figure 3c). The Pearson correlation test between the TPH/PaH expression and serotonin content was positive for the embryos (r = 0.926, *P* = 0.0044). A similar result with lower expression was observed for the 48-hour embryos and 1-week juveniles (data not shown).

NvHIOMT had increased expression at the beginning of the light period and had significantly lower expression during the dark period for adult animals (Figure 3b, light-gray line). This pattern correlated with the pattern of melatonin production observed, with a 4-hour advance of the HIOMT gene expression relative to the melatonin peak determined with ELISA (Pearson correlation test r value = 0.696, *P* = 0.042). For animals in constant darkness, HIOMT expression was near constant during the 24-hour period (Figure 3b, black line). Expression of the HIOMT gene in the 2-week-old juveniles also was similar to that pattern of serotonin and melatonin production (Figure 3d), with a peak 6 hours after lights on (ZT = 6). The Pearson correlation test between the HIOMT expression and melatonin content was positive for the embryos (r = 0.931, *P* = 0.0108). A similar result was observed for the 48-hour embryos and 1-week juvenile (data not shown).

To further validate the serotonin and melatonin assays, we performed enzymatic assays for tryptophan hydroxylase and hydroxyindol-O-methyltransferase for adults from ZT = 8 and ZT = 20. These time periods showed activity for each enzyme at both time points (Table 1). Highest activity was measured at the ZT = 8, the same point of the melatonin peak. Enzymatic assays were also performed with samples from embryos from ZT = 6. The measured activity of tryptophan hydroxylase was 38.94 ± 7.32 picomoles per 200 mg of protein per hour (n = 4) and 61.51 ± 0.74 picomoles per 200 mg of protein per hour (n = 4) for hydroxyindol-O-methyltransferase.

**Table 1:** Measures of activity of tryptophan hydroxylase (TPH) and hydroxyindol-O-methyltransferase (HIOMT) with samples of the animals at Zeitgeber time (ZT) = 8 (that is, 8 hours after lights on) and ZT = 20 (that is, 8 hours after lights off)

**Enzymatic assay/Time point**	**ZT = 8**	**ZT = 20**
TPH	78.94 ± 7.32	58.96 ± 4.73
HIOMT	91.71 ± 0.80	43.51 ± 0.99

Values are mean ± SEM for four samples per assay. Activity expressed in picomoles per 200 mg of protein per hour. ZT20 values are statistically different from ZT = 8; *P* <0.05.

### Developmental expression of the circadian clock genes and generation of rhythmicity

**Figure 4:**
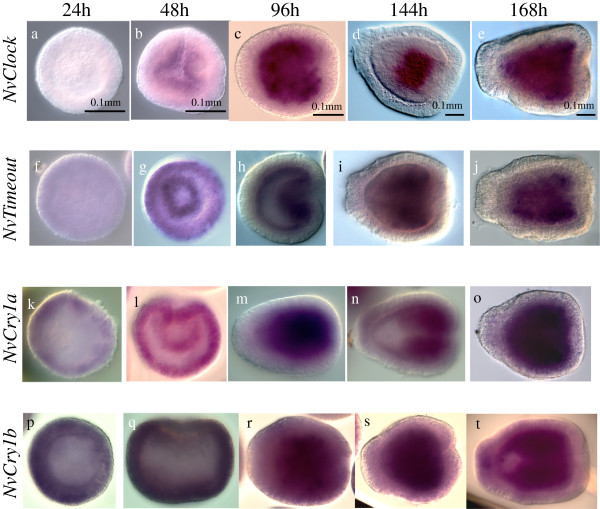
**Temporal expression dynamics of *NvClock, NvTimeout, NvCry1a *and *NvCry1b *genes by whole mount in situ hybridization.** The stages for each gene represent embryos 24 - **(a, f, k, p)**, 48- **(b, g, l, q)** 96- **(c, h, m, r)**, 144- **(d, i, n, s)** and 168- **(e, j, o, t)** hours post-fertilization.

To characterize the potential role of *N. vectensis* clock genes in embryonic development, we examined the spatial and temporal expression dynamics of those genes by whole mount in situ hybridization. Figure 4 shows the expression patterns of *NvClock, NvTimeout, NvCry1a* and *NvCry1b* (*NvCycle* reported in [43]). Diffuse expression of *NvClock* begins at blastula stage (Figure 4a). Expression is concentrated in the oral pole during late blastula (Figure 4b) and then remains restricted to the endoderm during the gastrula (Figure 4c) and late planula (Figure 4d-e) stages of development. Expression appeared to be uniform in all cells of the endoderm and did not show the ‘salt and pepper’ pattern characteristic of cells in the endodermal nervous system [44].

*NvTimeout* showed a pattern similar to *NvClock*, weak expression at the blastula stage (Figure 4f) with expression intensifying at the oral pole during invagination (Figure 4g). After this stage, expression is then restricted to the endoderm, during early (Figure 4h) and late planula (Figure 4i-j).

The cryptochrome genes *NvCry1a* and *NvCry1b* show similar patterns. The expression begins at the blastula stage diffusely in future ectoderm (Figure 4k,p). During invagination of prospective endoderm at gastrulation, expression begins to be expand to the endoderm for *NvCry1a* (Figure 4l), and over time restricts its expression to become more restricted to the endoderm in later stages (Figure 4m-o). A similar pattern of expression is observed for *NvCry1b*; predominantly ectodermal during gastrulation (Figure 4q) but switching to endoderm during later gastrula stages (Figure 4r). In the planula stages, the expression is more concentrated in the endoderm, but faint expression was evident in ectodermal tissues (Figure 4s-t).

We performed qPCR assays on the clock genes to investigate the generation of the rhythmicity during *N. vectensis* embryogenesis. *NvClock*, *NvTimeout*, *NvCry1a* and *NvCry1b*, at six hour time points over a 24-hour period (that is, 6, 12, 18, and 24-hours post-fertilization (blastula)), 48-hours (late blastula), 1-week (late planula) and 2-weeks (early polyp with tentacle buds) post-fertilization.

**Figure 5:**
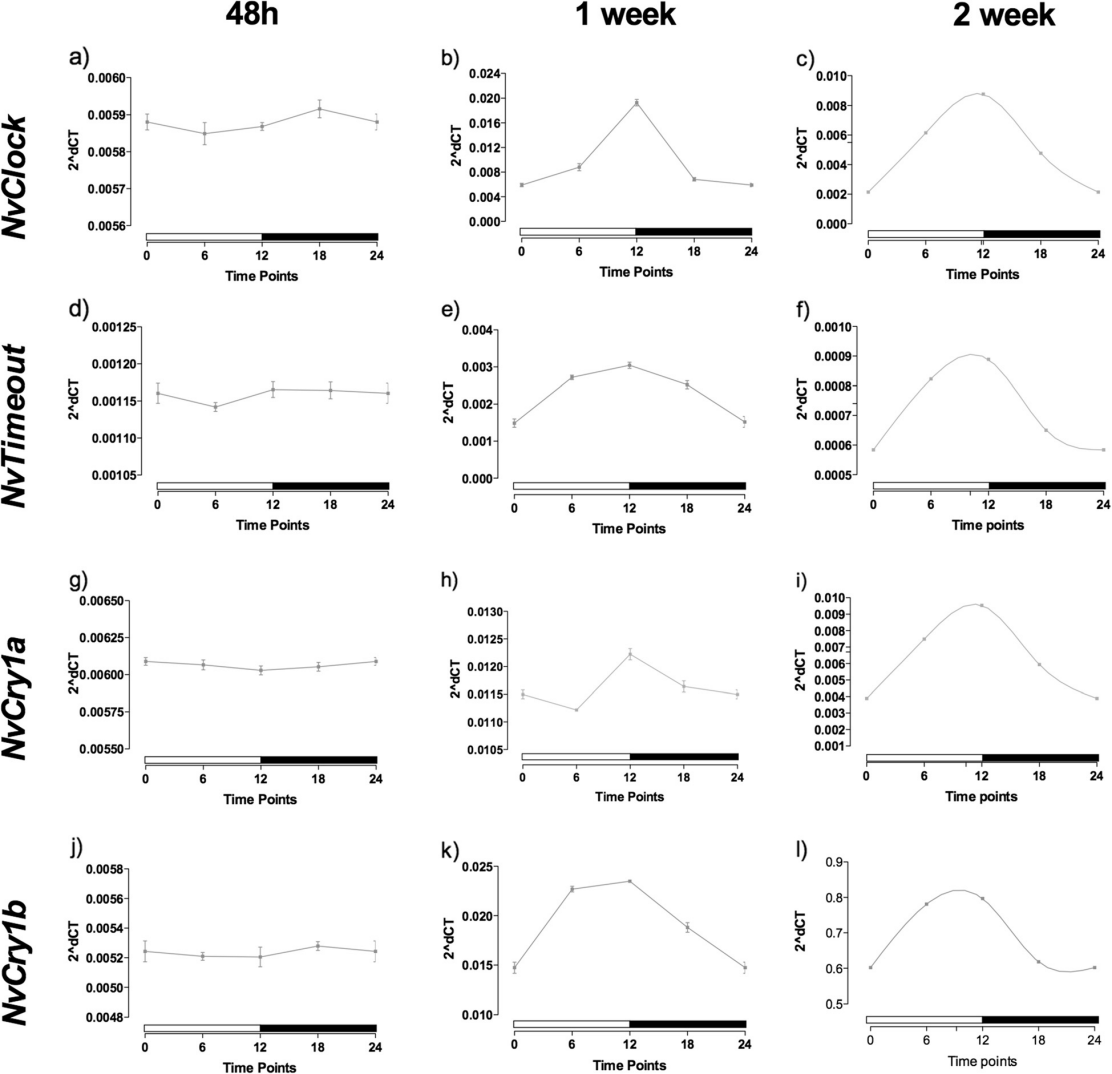
**Relative mRNA expression of *NvClock*, *NvTimeout*, *NvCry1a *and *NvCry1b *in embryos of *Nematostella vectensis *normalized by the gene to the ribosomal protein P0.** Samples from 48-h, 1-week, and 2-weeks post-fertilization. Each point represents five individuals/samples.

Up to the 48-hours post-fertilization, there was no clear rhythm of expression of *NvClock* (Figure 5a). One-way ANOVA test showed no difference in expression levels between any of the time points (*P* = 0.4179). At 1-week post-fertilization, there was evidence of a rhythm (Figure 5b), with a peak in the transition light–dark (ZT = 12), that was confirmed by the one-way ANOVA (*P* <0.0001). However, the cosinor test was not valid, indicating that even that there was variation between time points that did not follow a 24-hour period. In the 2-week polyps, however, there was a clear circadian pattern of expression, with variation confirmed by the one-way ANOVA test (*P* <0.0001), and the cosinor test was valid, (*P* = 0.048), indicating that this gene had acquired a circadian pattern of expression (Figure 5c).

For the *NvTimeout*, again there was no rhythmicity in expression in 48-hour embryos (Figure 5d), confirmed by the one-way ANOVA (*P* = 0.7247). In the 1-week polyps, however, it was evidence of rhythmic expression (Figure 5e). The one-way ANOVA showed that expression was significantly different (*P* = 0.0008) and the cosinor test was valid (*P* = 0.045), revealing that the gene already had a circadian pattern of expression (graph not shown). Rhythmic expression was maintained in the 2-week polyps (Figure 5f), where the cosinor model was also valid (*P* = 0.025).

The gene *NvCry1a* had a pattern similar to *NvClock*: no rhythm of expression in the 48-hour embryos (Figure 5g, *P* = 0.3745), but with a difference between the time points in the 1-week embryos (*P* <0.0001), without validation from the cosinor (Figure 5h). At 2 weeks, the polyps had a circadian pattern of expression of the gene (Figure 5i), with validation of the one-way ANOVA (*P* <0.0001) and from the cosinor (*P* = 0.003). The pattern for *NvCry1b*was similar to *NvTimeout*. In 48-hour embryos, there was no rhythmicity in expression (Figure 5j), which was confirmed by the one-way ANOVA, which showed no difference in expression between time points (*P* = 0.928). In the 1-week polyps, however, we detected a rhythm (Figure 5k) with significant differences in expression over time (*P* = 0.0004) and a significant cosinor result (*P* = 0.0072, graph not shown). This circadian pattern of expression was continued in the 2-week-old polyps (Figure 5l), where again the one-way ANOVA showed difference between the points (*P* <0.0001) and the cosinor model was valid (*P* = 0.0052).

### Clock attenuation and melatonin-initiated clock gene activation

We analyzed transcription of clock genes in adults to determine how their expression was altered by removal of a light cycle and investigate if the addition of exogenous melatonin alters the expression of these genes in the absence of a light cue. Previous work showed that 30 days of constant darkness appears to result in the loss of rhythmicity in the same clock genes when assayed with qPCR [35].

**Figure 6:**
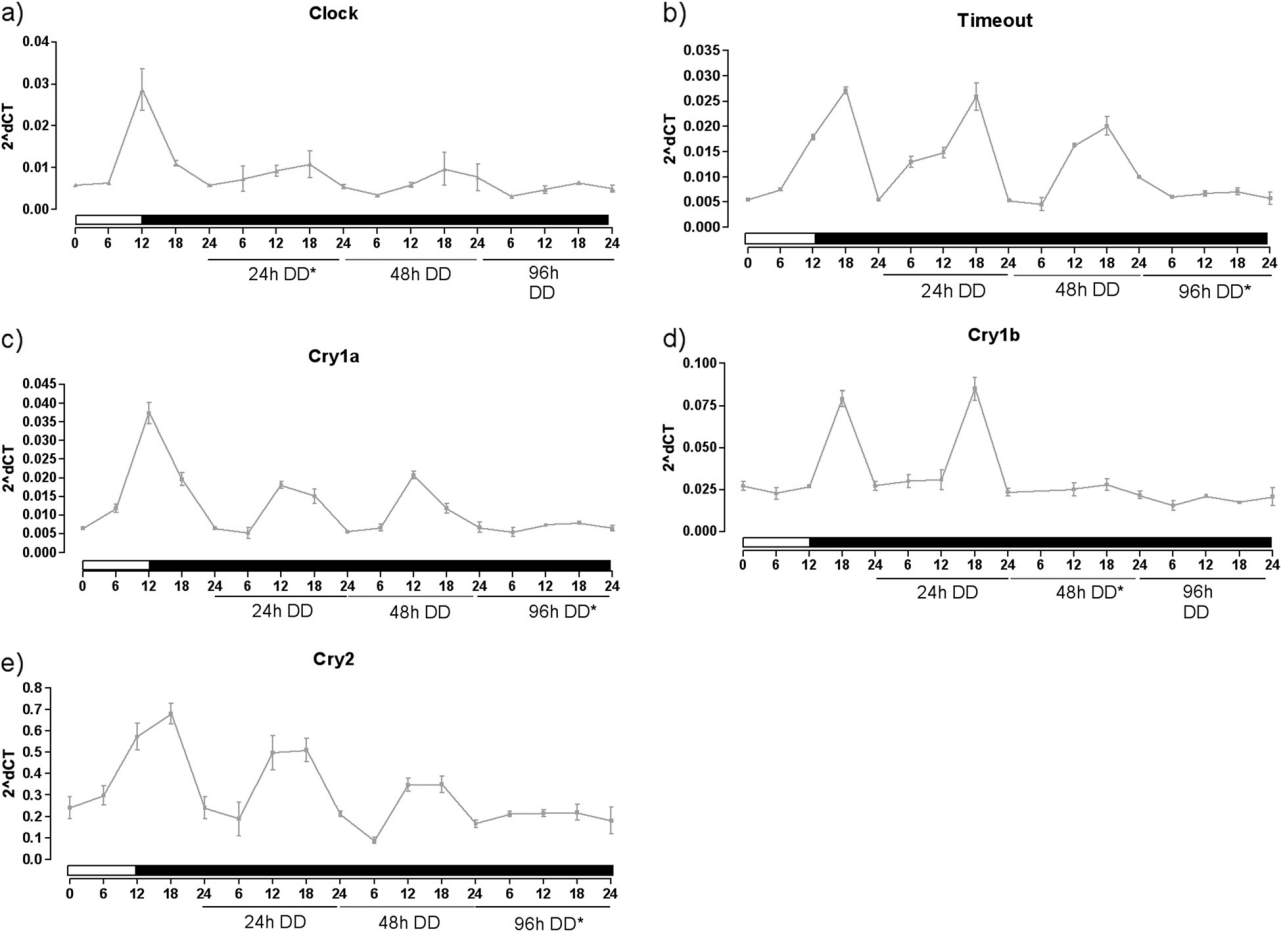
**Relative mRNA expression of Clock Genes in *Nematostella vectensis.*** Data of **a)***Clock*, **b)***Timeout*, **c)***Cry1a*, **d)** Cry1b and **e)***Cry2* in individuals of *Nematostella vectensis* in light–dark cycle and 24 h, 48 h or 96 h after the animals were moved to the constant darkness. *indicates the moment where the one-way ANOVA test indicates that there is no more difference between the time points within the 24-h cycle (*P* >0.05.) Each point represents five individuals/samples.

First, in order to investigate how rapidly the *N. vectensis* clock genes lost their rhythmicity in adults removed from the entraining light cue, we moved animals entrained in the normal 12:12 light–dark cycle to constant darkness and sampled individuals at 24, 48 and 96-hours. *NvClock*, lost rhythm in the first 24 hours of constant darkness, with no difference in the points along the period (Figure 6a, one-way ANOVA, *P* = 0.1880). *NvTimeout, NvCry1a* and *NvCry2* maintained rhythmic expression for longer than *NvClock*, but by 96 hours all showed expression profiles lacking oscillations (one-way ANOVA, *P* = 0.2532 for *NvTimeout*, 0.1081 for *NvCry1a* and 0.4186 for *NvCry2*, Figure 6b,c and d). *NvCry1b* lost evidence of rhythmic expression at 48 hours (Figure 6e, one-way ANOVA, *P* = 0.6111).

**Figure 7:**
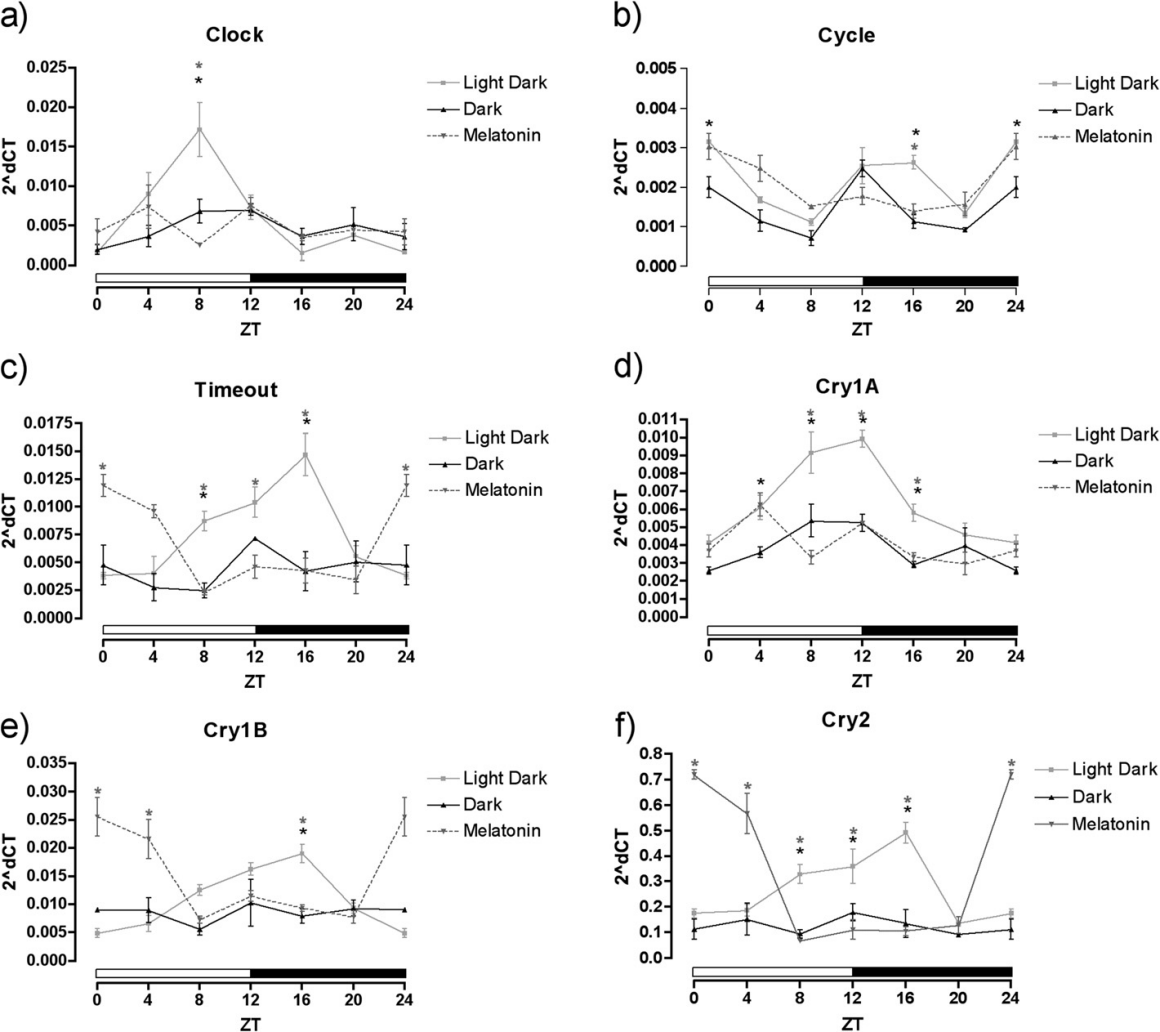
**Relative mRNA expression of Clock Genes in *Nematostella vectensis.*** Data of **a)***Clock*, **b)***Cycle*, **c)***Timeout*, **d)** Cry1a, **e)***Cry1b* and **f)***Cry2* in N*ematostella vectensis* kept in a light–dark (12:12 h), constant darkness, or constant darkness with melatonin supplementation (0.1 μM). Data normalized by the ribosomal protein P0. Each point represents five individuals. *Statistically different from light–dark with *P* <0.05.

**Figure 8:**
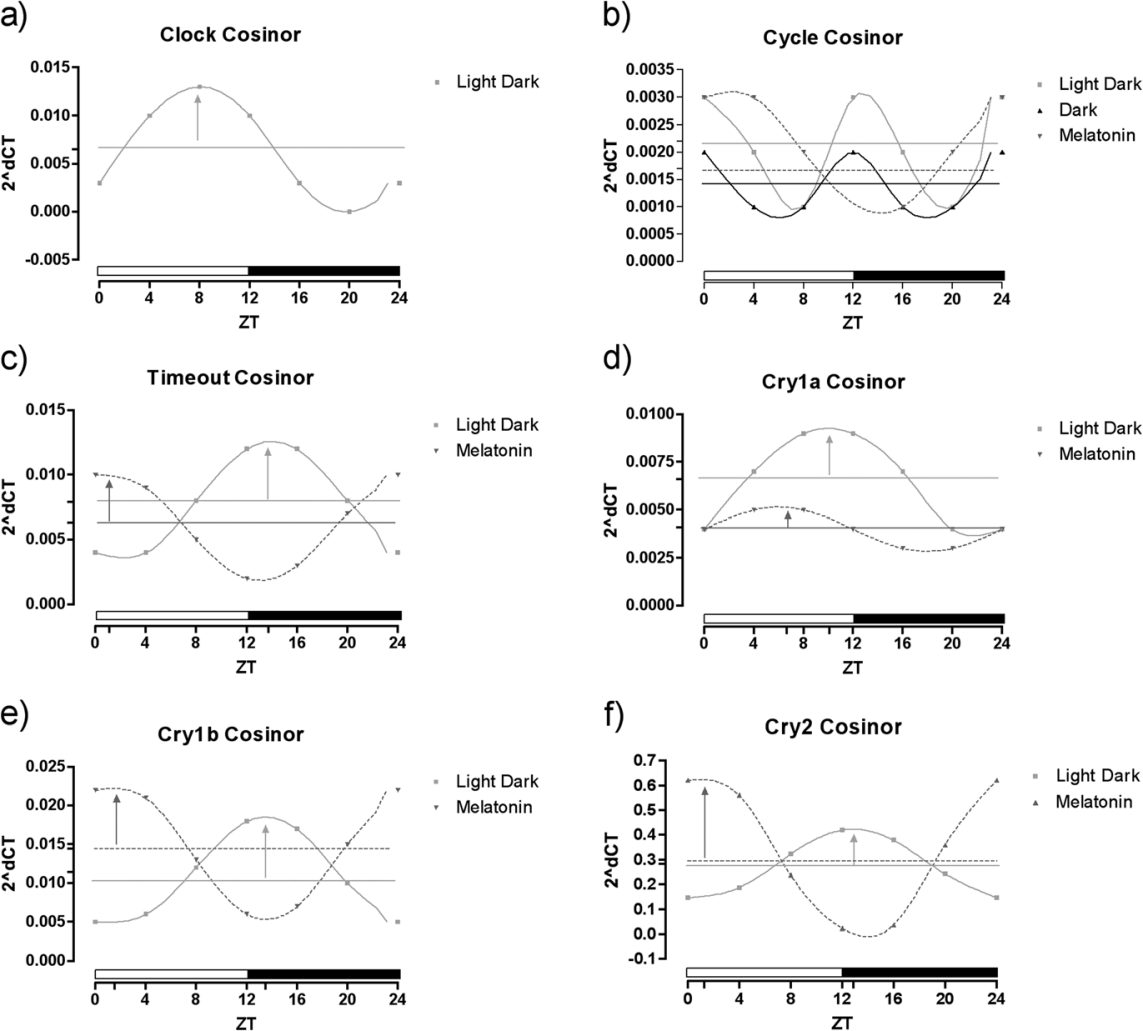
**Cosinor curves for the relative mRNA expression of Clock Genes in *Nematostella vectensis.*** Data of **a)***Clock*, **b)***Cycle*, **c)***Timeout*, **d)***Cry1a*, **e)***Cry1b* and **f)***Cry2* in individuals of *Nematostella vectensis* kept in a light–dark (12:12 h), constant darkness cycle, or constant darkness with melatonin supplementation (0.1 μM) normalized by the ribosomal protein P0, where a significant curve fit was obtained. The horizontal line, the mesor, represents the mean oscillation, and the vertical arrow, the acrophase, the time of occurrence of the daily-peak of melatonin. Each point represents five individuals/samples.

Next, we measured expression of these genes in *N. vectensis* adults cultured in darkness for 20 days (approximately 16 days after loss of rhythmic gene expression) and supplemented with melatonin at ZT = 12. Melatonin treatment did not alter the expression pattern of *NvClock*; gene expression lacked oscillations in expression similar to animals in constant darkness, which contrasts with increased expression of *NvClock* during light periods when animals are cultured on a diel cycle (Figure 7a) [38]. The cosinor test showed that the variation in the expression of *NvClock* had a 24-hour period only in animals in a normal light–dark cycle (Figure 8a).

The result observed for the gene *NvCycle* was interesting because altering the light cycle did not alter substantially the pattern of expression of the gene (Figure 7b). The time points were significantly different both between and within treatments (one-way ANOVA, *P* <0.005), which enabled us to do a cosinor test that was valid for all groups (Figure 8b).

*NvTimeout* showed a significantly different pattern of expression in the animals throughout the light–dark cycle, with a peak 3 hours after the transition to the dark period (Figure 7c). The one-way ANOVA test showed that the expression levels were different during the 24-hour period (*P* = 0.0006) for this group and for the melatonin-treated animals (*P* <0.0001) but not significantly different for the animals in constant darkness (*P* = 0.7985). The cosinor test showed that the gene had a circadian pattern of expression, but with a difference in the acrophase of almost 13 hours between the light–dark and the melatonin-treated animals (Figure 8c, Table 2).

**Table 2:** Cosinor parameters of the clock genes where a significant curve fit was obtained

**Parameter**	**Acrophase**		**Mesor**		**Amplitude**	
Gene	LD	Melatonin	LD	Melatonin	LD	Melatonin
*NvTimeout*	13.27 **±** 0.32	**0.04 ± 1.19**	0.0079 **±** 0.0008	**0.0063 ± 0.0011**	0.0049 **±** 0.001	0.0042 **±** 0.0015
*NvCry1a*	10.04 **±** 0.174	**6.24 ± 2.17**	0.0066 **±** 0.0003	**0.0041 ± 0.0005**	0.003 **±** 0.0003	**0.001 ± 0.0007**
*NvCry1b*	13.18 **±** 0.17	**1.2 ± 1.18**	0.011 **±** 0.02	**0.0144 ± 0.0023**	0.007 **±** 0.0008	0.0086 **±** 0.0031
*NvCry2*	12.32 **±** 1.13	**1.11 ± 1.04**	0.28 **±** 0.033	0.30 **±** 0.068	0.14 **±** 0.044	**0.343 ± 0.091**

‘LD’ refers to animals kept in a light–dark cycle. ‘Melatonin’ refers to animals kept in a constant darkness cycle with melatonin (0.1 μM) supplementation. Values are mean ± SEM for five samples per assay. Bold indicates significant difference.

The two Type I and single Type II cryptochromes from *N. vectensis* showed differential responses *NvCry1a* showed a similar expression pattern as *NvTimeout* (Figure 7d) with the cosinor model showing that the light–dark and the melatonin-treated animals showed significant 24-h rhythms that were lost in the constant darkness group (Figure 8d). *NvCry1b* showed circadian expression for the light–dark and melatonin-treated animals, but in phase opposition to one another (Figure 7e). The cosinor analysis showed rhythmic expression for both those groups, showing again the ability of melatonin to alter the pattern of expression of genes likely associated with the anemone circadian clock (Figure 8e). The pattern of the gene *NvCry2* was very similar to the one of *NvCry1b*, with phase opposition between the light–dark and the melatonin-treated animals and an expression pattern without oscillations for those in constant darkness (Figure 7f, 8f).

## Discussion

We provide multiple lines of empirical evidence (HPLC and ELISA) showing the presence of melatonin in the anthozoan *Nematostella vectensis* in different life stages, as well as enzymatic and transcription data suggesting conservation of portions of the melatonin synthesis pathway described in vertebrates. These data are consistent with previous papers that have described melatonin in anthozoan cnidarians as well as the diel oscillations in some species when exposed to light:dark cycles. We extend these previous observations to show that melatonin exposure could be important as a clock input regulating the expression of the genes that likely drive the cnidarian circadian clock in the absence of light cues.

### Patterns of melatonin synthesis in cnidarians

Previous research has shown variation in the seasonal and diel synthesis of melatonin in anthozoan cnidarians. Melatonin was first reported in the colonial anthozoan *Renilla köllikeri*[37]*,* where melatonin amounts fluctuated by season but not diel cycles. Melatonin was also at highest concentration in gametogenic regions. This work supported a role for melatonin in cnidarian seasonal reproduction but not circadian processes, which differs from bilaterians [8,14]. Subsequent studies in two other anthozoan species (*Actinia equina* and *Nematostella vectensis*) [38,39] showed that melatonin was also localized near or in gametogenic tissues. In addition, Roopin and Levy [38] showed that melatonin levels in *A. equina* vary on a diel cycle with peak concentration in early subjective night, but the oscillating expression dissipates upon exposure to constant darkness. These later results suggest that melatonin may serve a role in the circadian clock because cnidarians lose oscillations in gene expression when light cues are removed [35,43,45]. We show that melatonin concentration, as well as the genes in the melatonin synthesis pathway, have oscillating patterns in *N. vectensis*. The presence of nocturnal peaks of melatonin production in *N. vectensis* is similar to peak production of melatonin in diverse species. Nocturnal peaks are present in all the vertebrates investigated thus far [46], as well as species of algae [8,47], flatworms [10] and insects, including *Drosophila *[48]*.* Combined, these results suggest that melatonin has diel patterns in anthozoans, which are likely related to the circadian clock (see below).

We determined that TPH/PaH and HIOMT, central components of the melatonin pathway, are present and expressed in *N. vectensis*. Both enzymes have already been described in different groups of invertebrates, including flatworms [49], sea urchins [50] and cnidarians [51] and the activity of the enzymes in mollusks [52] and arthropods [53-55]. We found a correlation between the rhythm of the transcription of TPH/PaH and production of serotonin (product of TPH in vertebrates) and HIOMT and production of melatonin (product of HIOMT), which provides consistent evidence that these enzymes participate in the melatonin pathway. The fact that we were also able to measure the activity of the enzymes gives addition support for the presence of a vertebrate-like melatonin pathway in cnidarians.

Our work presented here is the first to show melatonin synthesis in a cnidarian during invertebrate embryogenesis. There are few references in the literature of melatonin in embryos, showing its presence in fishes [29], birds [30] and mammals [56]. The measurement of a rhythm in the melatonin production during development indicates a regulated production of the hormone in response to light environment, instead of maternal deposition into maturing oocytes. In addition, because developmental stages lack gametogenic tissue, the role for melatonin in cnidarians includes non-reproductive functions in addition to the hypothesized role in gametogenesis reported in previous studies [37-39]. The melatonin pattern of production in the embryos resembles the adults, with peaks during the second half of the light phase. These data suggest that a circadian pattern is initiated in *N. vectensis* prior to the adult stage. However, the melatonin content has higher variation between these life cycle stages, with a peak value close to 26 picograms per milligram of protein in the 2-week-old embryos and around 6 picograms per milligram in adults. This difference may indicate either a higher production in the embryos or a higher degradation of melatonin in the adults.

### Melatonin as an input into the cnidarian circadian clock

The analysis of the genes likely involved in the circadian clock in the adults showed that 4 days of constant darkness were adequate to abolish the circadian expression for virtually all of them. Our data are nearly identical to the pattern previously observed, although that study kept *N. vectensis* for 30 days in constant darkness [35]; however, our results show that quenching of cyclical gene expression occurs within days of removal of light cues. Data from corals have similarly shown loss of the rhythmicity of some clock genes within 24 hours (*Acropora millepora *[57]) or 72 hours *(Favia fragum *[45]). Together, these data suggest that loss of rhythmic gene expression is characteristic of cnidarian clocks, in opposition to the classical description of the bilaterian clock, which is capable of maintaining rhythmicity even after several days in constant darkness [58-60].

We tested whether melatonin could re-synchronize the clock genes in individuals cultured in total darkness because work in mammals has shown that melatonin can serve as a potent molecule for circadian clock resetting [61,62]. Previous studies in mammals showed that a short melatonin pulse (2 hours) is not enough to alter the expression of clock-related genes [63]. We observed that the addition of melatonin in 12-hour pulses results in oscillations in expression of *N. vectensis* circadian clock genes, supporting a hypothesis that the interaction of melatonin and clock genes appeared early in the evolution of the animals.

The differences in the timing of peak melatonin expression observed for clock genes between animals in the light:dark cultures and constant darkness with melatonin supplementation are likely due to the timing of melatonin introduction. Animals in a light–dark cycle showed increasing melatonin beginning at ZT = 8 that was sustained into subjective night. We added melatonin to cultures of animals in constant darkness at the subjective transition to the dark phase (that is, ZT = 12), which could explain the temporal shift in gene expression. In vivo melatonin is produced in a progressive way, with low concentrations in the beginning of the day, which increase during subjective day and peak during the night phase of the light:dark cycle. In our treatment, we added melatonin at the beginning of subjective night (ZT = 12) to coincide with the highest concentration of melatonin. Future experiments that manipulate the timing of melatonin supplementation would provide an additional empirical test for how melatonin additions may shift transcription of genes involved in the circadian clock and how the timing of addition relates to the endogenous melatonin levels.

Considering the potential interplay between melatonin and clock genes, it is difficult to postulate whether the changes we observed in the melatonin production in the animals in constant darkness are a product of the disruption of the rhythmicity of the clock genes, or vice versa. Manipulations to block the melatonin production with morpholinos to TPH/PaH or HIOMT, or to knockdown the expression of the clock genes themselves, could be useful to better understand the interaction between the two factors. These studies would test for conserved functions for the role of hormones in the transcription-translation feedback loop of the cnidarian circadian clock.

#### ***Additional potential function(s) of melatonin in Nematostella vectensis***

Although melatonin concentration peaks in the dark period of light:dark cycles, the presence of melatonin in the light period could also have functions that extend beyond its role in a circadian clock. Melatonin can function as an antioxidant capable of reducing reactive species [64]. These oxidative species are not only produced by endogenous sources, like metabolism, but also by exogenous ones, such as pollutants (for example, toxic metals) and ultraviolet radiation [65,66]. *N. vectensis* lives in the shallow intertidal zone, with potentially high exposure to ultraviolet radiation [67], and thus melatonin may serve a similar, additional function as an antioxidant. Considering that during embryogenesis there is a high metabolism rate, (for example, high mitotic activity) [68], melatonin production could also help mitigate the production of free radicals during these stages.

Another possible function for the melatonin observed in the early embryos could be its participation in a ‘diffuse neuroendocrine system’ [69], acting at early developmental stages before differentiation of the nervous system. In this way, serotonin, which we demonstrate to be present in the *N. vectensis* embryos, could have a function in the regulation of basic developmental processes (cell migration, differentiation and proliferation). A role for serotonin in development has been reported for sea urchin, mouse, and other vertebrate embryos [70-73]. Melatonin has also already been reported to act to stimulate cell proliferation and accelerate development in zebrafish embryos [74].

### Ontology of the cnidarian circadian clock and relationship to melatonin synthesis

Quantitative and spatial analysis of clock gene expression during development indicates that their expression begins early in the development that precedes oscillating expression in response to environmental cues, similar to zebrafish. In situ expression analysis of these circadian genes showed that all genes are expressed broadly in the endoderm post-gastrulation. The two Type I cryptochrome paralogs *Cry1a* and *Cry1b* were expressed prior to gastrulation with general expression in the blastula. The lack of specific expression in any region of the endoderm precludes any clear hypotheses regarding potential developmental functions of these genes. Previous work describing expression of circadian clock genes in the development of zebrafish has suggested that these genes may function in cell cycle control prior to a role in circadian clock function in later stages. A similar function may be present in *N. vectensis*.

Two genes (*Timeout* and *Cry1b*) are transcribed in a circadian fashion in the 1-week-old embryos, and at two weeks, all the clock genes we evaluated were being expressed in a circadian rhythm, with a similar pattern to that observed in the adults. These results suggest that the circadian clock may not begin oscillating until the polyp stage. Interestingly, we observed melatonin concentrations fluctuated during embryogenesis, despite the lack of oscillating expression of the circadian clock. The difference in timing of oscillatory behavior of melatonin and circadian clock may indicate a non-circadian role for melatonin in development, potentially in responding to oxidative stress. Future research measuring behavioral entrainment of larval stages will be necessary to determine in *N. vectensis* larvae have diel activity patterns like those described in adult polyps [75].

## Conclusions

Our results support an ancient role for melatonin in the circadian behavior of animals by showing cyclic expression of this hormone under diel conditions, both in adults and in embryos. We also show light-dependent oscillations in gene expression in the melatonin synthesis pathway, and in activity of those enzymes, indicating that the melatonin pathway of production is likely conserved since the cnidarian-bilaterian ancestor. Our work is pioneering in showing melatonin initiating expression of circadian clock genes in the cnidarian *N. vectensis,* an event only showed before in mammals, supporting a hypothesis that the interaction of melatonin and clock genes appeared early in the evolution of the animals.

During development, melatonin shows oscillations during embryogenesis when developmental stages experience diel lighting conditions; however, the circadian clock genes show no evidence of rhythmicity, despite having specific spatial expression in the endoderm. We suggest that melatonin in these early stages can have functions to mitigate the production of free radical [68] or participate in potential neuroendocrine signaling [69]. The differences in expression of melatonin and the circadian clock gene network in the adult stage when compared with developmental stages of *N. vectensis* suggests new research directions to characterize stage-specific mechanisms of circadian clock function in animals.

## Abbreviations

AANAT: arylalkylamine N-acetyltransferase; BCP: 1-bromo-3-chloropropane; D: dark; HIOMT: hydroxyindol-O-methyltransferase; HPLC: high performance liquid chromatography; L: light; NAS: N-acetylserotonin; PaH: phenylalanine hydroxylases; SCN: suprachiasmatic nucleus; TPH: tryptophan hydroxylase; ZT: zeitgeber time; 5HTP: 5-hydroxytryptophan.

## Competing interests

The authors declare that they have no competing interests.

## Authors’ contributions

RP performed most of the experiments and drafted the article. RP, AMR and MQM performed the analysis of the data. RP and YP performed the experiments with embryos. RP and SCA performed the enzyme activity assays. RP, AMR, MQM, ACM and JCN participated in the design of the study. AMR and MQM helped to draft the manuscript. All authors read and approved the final manuscript.

## Supplementary Material

Additional file 1 Primers used in the qPCR reactions. Click here for file

Additional file 2 **Chromatograms of a melatonin standard (black line) and of a *Nematostella vectensis *sample (blue line).** The chromatograms show the same pattern, with the same retention time for melatonin, validating the assay. Click here for file

Additional file 3 **Formatted Alignments of the sequences of TPH gene in *Nematostella vectensis* genome, our clone and the qPCR amplified fragment.** Click here for file

Additional file 4 **Formatted Alignments of the sequences of HIOMT gene in *Nematostella vectensis* genome, our clone and the qPCR amplified fragment.** Click here for file

Additional file 5 Phylogenetic analyses of TPH gene. Click here for file

Additional file 6 Phylogenetic analyses of HIOMT gene. Click here for file
