# Supplementary material for: Developmental and light-entrained expression of melatonin and its relationship to the circadian clock in the sea anemone Nematostella vectensis
Source: EvoDevo. 2014 Aug 14;5:26. doi: 10.1186/2041-9139-5-26 (PMC4169136; doi:10.1186/2041-9139-5-26)
Supplement: Additional file 1 — Primers used in the qPCR reactions. [file 2041-9139-5-26-S1.pdf]

Supplementary Information 1:

Primers information:

- Clock, 5’-TCGGCTCCCCGAGTCTAGCG-3’(sense) and 5’-GCATCGCGGCGAACCCAGTA-3’ (antisense), 59.8ºC, 110bp, accesion number XP_001639742
- Cycle, 5’-TGTCTCGTGGCCGTCGGAAG-3’(sense) and 5’-GGCGACATCCGGTCGGGAAAA-3’ (antisense), 59ºC, 72bp, accesion number XP_001624731
- Timeout, 5’-GTGCCTTCGCAGGTGGGACG-3’(sense) and 5’-GGCTTTCGTTGTCCGACTCGCT-3’ (antisense), 60ºC, 96bp, accesion number XP_001641000
- Cry1a, 5’-GCGGCATGGCAAGACAGGCT-3’(sense) and 5’-CACCCAATGGACTGCCGGGC-3’ (antisense), 60.3ºC, 99bp, accesion number XP_001631029
- Cry1b, 5’-TCGGACCGGACCATGCTGACA-3’(sense) and 5’-TCGTCGCAAAGCTTCGGGCT-3’ (antisense), 59.6ºC, 122bp, accesion number XP_001632849
- Cry2, 5’-AGCTCGCCGTAAAAGGGCGG-3’(sense) and 5’-ATATAAGCGCCTGCACCCGCG-3’ (antisense), 59.8ºC, 75bp, accesion number XP_001623146
- TPH: 5’-AAAAACTCGCCACGCTCTACTGG-3’(sense) and 5’-CGGTCAAGCAATACTGTAACTCCCC-3’ (antisense), 56.4ºC, 123bp, accesion number XM_001623795.1
- HIOMT: 5’-CGAGATTTGATTGCGTTCACCG-3’(sense) and 5’-AAAACAGCCAGTTCCTCCTCCAAG-3’ (antisense), 59ºC, 107bp, accesion number XM_001627179.1
- Ribosomal protein P0: 5’-GGCTTCGTCTTCACCAAGGAGGAG-3’(sense) and 5’-CCAGCAGGGACAAACACATCAATA-3’ (antisense), 60.5ºC, 117bp, accesion number

XM_001626244.1
