# Supplementary material for: Developmental and light-entrained expression of melatonin and its relationship to the circadian clock in the sea anemone Nematostella vectensis
Source: EvoDevo. 2014 Aug 14;5:26. doi: 10.1186/2041-9139-5-26 (PMC4169136; doi:10.1186/2041-9139-5-26)
Supplement: Additional file 2 — Chromatograms of a melatonin standard (black line) and of a Nematostella vectensis sample (blue line). The chromatograms show the same pattern, with the same retention time for melatonin, validating the assay. [file 2041-9139-5-26-S2.pdf]

7 NV1

Sample Name:	**NV1**	*Injection Volume:*	**40,0**
Vial Number:	**GB10**	*Channel:*	**ECD_1**
Sample Type:	**unknown**	*Wavelength:*	**n.a.**
Control Program:	**5041 coluna menor melatonina_750mV**	*Bandwidth:*	**n.a.**
Quantif. Method:	**melatonina coluna menor**	*Dilution Factor:*	**1,0000**
Recording Time:	**27/8/2012 12:10**	*Sample Weight:*	**1,0000**
Run Time (min):	**10,01**	*Sample Amount:*	**1,0000**

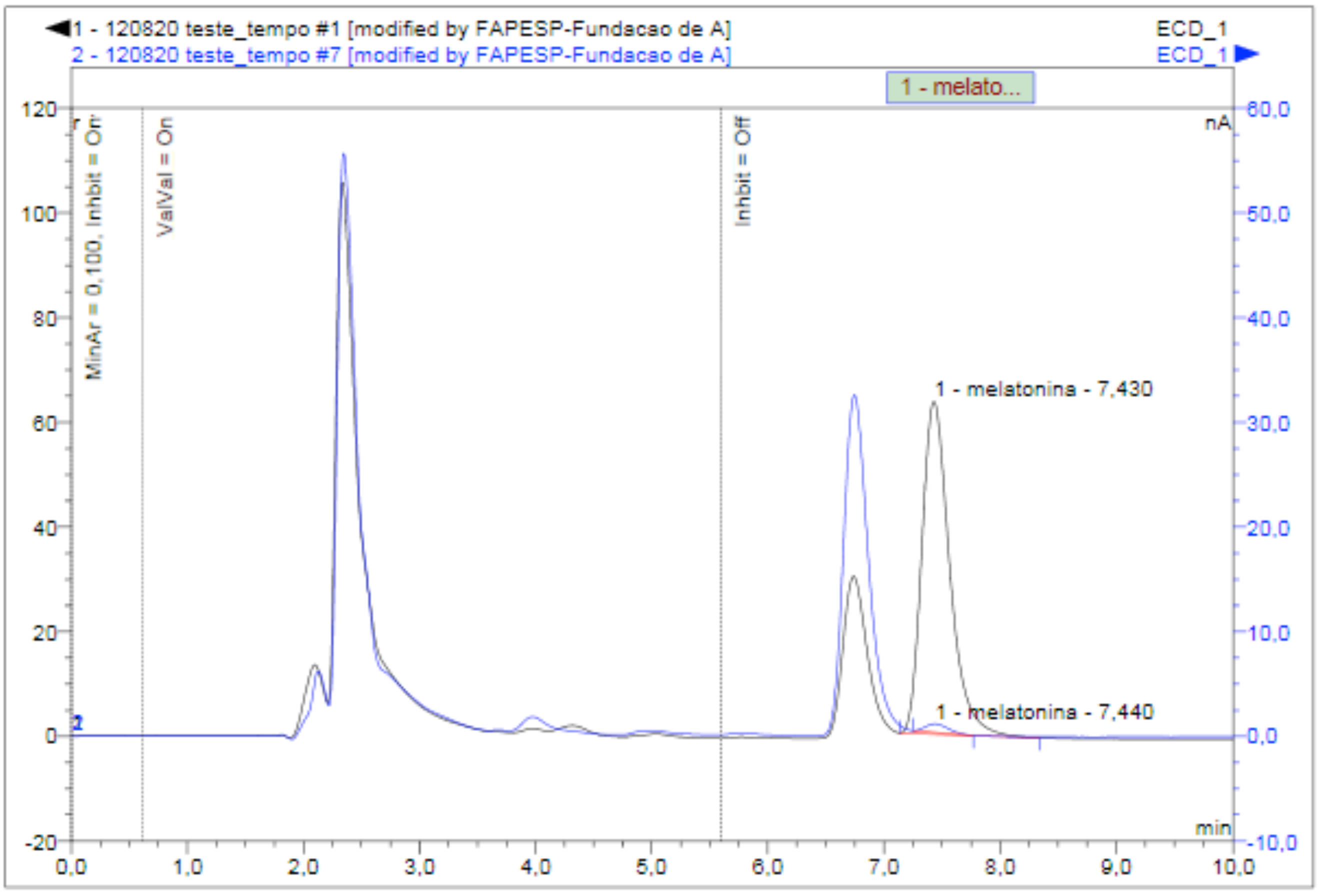

No.	Ret.Time min	Peakname min	Height nA	Width min	Type	Resol. (EP)	Asym. (EP)	Plates (EP)
1	7,440	melatonina	0,796	0,364	BMB*	n.a.	1,27	6423
**Average:**			0,796	0,364		n.a.	1,27	6423

Chromatograms of a melatonin standard (black line) and of a *Nematostella vectensis* sample (blue line). The chromatograms show the same pattern, with the same retention time for melatonin, validating the assay.
