# Supplementary material for: Developmental and light-entrained expression of melatonin and its relationship to the circadian clock in the sea anemone Nematostella vectensis
Source: EvoDevo. 2014 Aug 14;5:26. doi: 10.1186/2041-9139-5-26 (PMC4169136; doi:10.1186/2041-9139-5-26)
Supplement: Additional file 3 — Formatted Alignments of the sequences of TPH gene in Nematostella vectensis genome, our clone and the qPCR amplified fragment. [file 2041-9139-5-26-S3.pdf]

Formatted Alignments

```
                         10        20        30
tph_nematostella GTGTGTTTCTTTGAGTGAGGAAAATGCACG
tph_qpcr         ------------------------------
tph_cloned       --------CTTTGAGTGAGGAAAATGCACG
                 GTGTGTTTCTTTGAGTGAGGAAAATGCACG

                         40        50        60
tph_nematostella CTGTAAGGCGCAAAAGAACCTACGAAATCG
tph_qpcr         ------------------------------
tph_cloned       CTGTAAGGCGCAAAAGAACCTACGAAATCG
                 CTGTAAGGCGCAAAAGAACCTACGAAATCG

                         70        80        90
tph_nematostella AGCCGGACCATGGCCCTGCTAAGAAGCATG
tph_qpcr         ------------------------------
tph_cloned       AGCCGGACCATGGCCCTGCTAAGAAGCATG
                 AGCCGGACCATGGCCCTGCTAAGAAGCATG

                        100       110       120
tph_nematostella AGGGCGAATCAGAAAAGAAAGCTACCACGG
tph_qpcr         ------------------------------
tph_cloned       AGGGCGAATCAGAAAAGAAAGCTACCACGG
                 AGGGCGAATCAGAAAAGAAAGCTACCACGG

                        130       140       150
tph_nematostella TAGTGTTTTCCCTGAATGAGGAGGTTGGCG
tph_qpcr         ------------------------------
tph_cloned       TAGTGTTTTCCCTGAATGAGGAGGTTGGCG
                 TAGTGTTTTCCCTGAATGAGGAGGTTGGCG

                        160       170       180
tph_nematostella CGTTGGCCAGAGCTTTGAAATTATTTGAGG
tph_qpcr         ------------------------------
tph_cloned       CGTTGGCCAGAGCTTTGAAATTATTTGAGG
                 CGTTGGCCAGAGCTTTGAAATTATTTGAGG

                        190       200       210
tph_nematostella ATCACCATGTGAATATGACGCACATTGAAT
tph_qpcr         ------------------------------
tph_cloned       ATCACCATGTGAATATGACGCACATTGAAT
                 ATCACCATGTGAATATGACGCACATTGAAT

                        220       230       240
tph_nematostella CCCGCCCGTCCAAGGCATGCAAGACCCGCT
tph_qpcr         ------------------------------
tph_cloned       CCCGCCCGTCCAAGGCATGCAAGACCCGCT
                 CCCGCCCGTCCAAGGCATGCAAGACCCGCT

                        250       260       270
tph_nematostella ATGATTTCTTCGCAGACTGTGAGGGGCTGC
tph_qpcr         ------------------------------
tph_cloned       ATGATTTCTTCGCAGACTGTGAGGGGCTGC
                 ATGATTTCTTCGCAGACTGTGAGGGGCTGC

                        280       290       300
tph_nematostella ATGGTGCTAGTCTGAATCACTTTGTTGATG
tph_qpcr         ------------------------------
tph_cloned       ATGGTGCTAGTCTGAATCACTTTGTTGATG
                 ATGGTGCTAGTCTGAATCACTTTGTTGATG

                        310       320       330
tph_nematostella CACTAAAGAAGAGAGCCGTCAATATTACTG
tph_qpcr         ------------------------------
tph_cloned       CACTAAAGAAGAGAGCCGTCAATATTACTG
                 CACTAAAGAAGAGAGCCGTCAATATTACTG

                        340       350       360
tph_nematostella TTCTATCTGATGATAAGGGCTCAGTGCCAT
tph_qpcr         ------------------------------
tph_cloned       TTCTATCTGATGATAAGGGCTCAGTGCCAT
                 TTCTATCTGATGATAAGGGCTCAGTGCCAT

                        370       380       390
tph_nematostella GGTTCCCCCGCAAGATTTCTGATCTGGACA
tph_qpcr         ------------------------------
tph_cloned       GGTTCCCCCGCAAGATTTCTGATCTGGACA
                 GGTTCCCCCGCAAGATTTCTGATCTGGACA

                        400       410       420
tph_nematostella AGTTTGCTGACAGAGTGCTGAGCTATGGAG
tph_qpcr         ------------------------------
tph_cloned       AGTTTGCTGACAGAGTGCTGAGCTATGGAG
                 AGTTTGCTGACAGAGTGCTGAGCTATGGAG

                        430       440       450
tph_nematostella CTGAGCTAGACTCAGACCATCCTGGCTTTA
tph_qpcr         ------------------------------
tph_cloned       CTGAGCTAGACTCAGACCATCCTGGCTTTA
                 CTGAGCTAGACTCAGACCATCCTGGCTTTA

                        460       470       480
tph_nematostella CAGACCAAGTATACAGAGCCAGACGAAAGG
tph_qpcr         ------------------------------
tph_cloned       CAGACCAAGTATACAGAGCCAGACGAAAGG
                 CAGACCAAGTATACAGAGCCAGACGAAAGG

                        490       500       510
tph_nematostella AGTTTGCTGACATTGCCTTCAAGCACAAAC
tph_qpcr         ------------------------------
tph_cloned       AGTTTGCTGACATTGCCTTCAAGCACAAAC
                 AGTTTGCTGACATTGCCTTCAAGCACAAAC

                        520       530       540
tph_nematostella ACGGTGAAATTATTCCACAGGTCACTTATA
tph_qpcr         ------------------------------
tph_cloned       ACGGTGAA-TTATTCCACAGGTCACTTATA
                 ACGGTGAAATTATTCCACAGGTCACTTATA

                        550       560       570
tph_nematostella CAGATATCGAGATTGCAACCTGGGATAAAG
tph_qpcr         ------------------------------
tph_cloned       CAGATATCGAGATTGCAACCTGGGATAAAG
                 CAGATATCGAGATTGCAACCTGGGATAAAG

                        580       590       600
tph_nematostella TGTTCACAGAGCTGACCAAGTTGTATCCTA
tph_qpcr         ------------------------------
tph_cloned       TGTTCACAGAGCTGACCAAGTTGTATCCTA
                 TGTTCACAGAGCTGACCAAGTTGTATCCTA

                        610       620       630
tph_nematostella CCCATGCTTGCCGTGAACATAACTTTGTCT
tph_qpcr         ------------------------------
tph_cloned       CCCATGCTTGCCGTGAACATAACTTTGTCT
                 CCCATGCTTGCCGTGAACATAACTTTGTCT

                        640       650       660
tph_nematostella GGCCATTGCTAGTGCAAAATTGTGGTTACA
tph_qpcr         ------------------------------
tph_cloned       GGCCATTGCTAGTGCAAAATTGTGGTTACA
                 GGCCATTGCTAGTGCAAAATTGTGGTTACA

                        670       680       690
tph_nematostella GACTTGGGAATATTCCTCAGCTTCAGGATG
tph_qpcr         ------------------------------
tph_cloned       GACTTGGGAATATTCCTCAGCTTCAGGATG
                 GACTTGGGAATATTCCTCAGCTTCAGGATG

                        700       710       720
tph_nematostella TCTCAATGTTTCTGAAAGAATGCACTGGGT
tph_qpcr         ------------------------------
tph_cloned       TCTCAATGTTTCTGAAAGAATGCACTGGGT
                 TCTCAATGTTTCTGAAAGAATGCACTGGGT

                        730       740       750
tph_nematostella TCACTTTACGGCCTGTGGCTGGACTTCTGT
tph_qpcr         ------------------------------
tph_cloned       TCACTTTACGGCCTGTGGCTGGACTTCTGT
                 TCACTTTACGGCCTGTGGCTGGACTTCTGT

                        760       770       780
tph_nematostella CATCCCGGGACTTCCTGGCGGGCCTGGCAT
tph_qpcr         ------------------------------
tph_cloned       CATCCCGGGACTTCCTGGCGGGCCTGGCAT
                 CATCCCGGGACTTCCTGGCGGGCCTGGCAT

                        790       800       810
tph_nematostella TCCGTGTGTTCCACAGTACTCAGTACATCC
tph_qpcr         ------------------------------
tph_cloned       TCCGTGTGTTCCACAGTACTCAGTACATCC
                 TCCGTGTGTTCCACAGTACTCAGTACATCC

                        820       830       840
tph_nematostella GTCACCCAACAAAACCCATGTACACCCCAG
tph_qpcr         ------------------------------
tph_cloned       GTCACCCAACAAAACCCATGTACACCCCAG
                 GTCACCCAACAAAACCCATGTACACCCCAG

                        850       860       870
tph_nematostella AGCCTGACGTTGTGCATGAGCTAATCGGTC
tph_qpcr         ------------------------------
tph_cloned       AGCCTGACGTTGTGCATGAGCTAATCGGTC
                 AGCCTGACGTTGTGCATGAGCTAATCGGTC

                        880       890       900
tph_nematostella ATGTGCCTCTGTTTGCTGATCCAGATTTCG
tph_qpcr         ------------------------------
tph_cloned       ATGTGCCTCTGTTTGCTGATCCAGATTTCG
                 ATGTGCCTCTGTTTGCTGATCCAGATTTCG

                        910       920       930
tph_nematostella CACAATTTAGTCAGGAAATTGGTCTTGCCT
tph_qpcr         ------------------------------
tph_cloned       CACAATTTAGTCAGGAAATTGGTCTTGCCT
                 CACAATTTAGTCAGGAAATTGGTCTTGCCT

                        940       950       960
tph_nematostella CCCTGGGAGCACCAGACGAGTGGGTGGAAA
tph_qpcr         -----------------------------A
tph_cloned       CCCTGGGAGCACCAGACGAGTGGGTGGAAA
                 CCCTGGGAGCACCAGACGAGTGGGTGGAAA

                        970       980       990
tph_nematostella AACTCGCCACGCTCTACTGGTTCACTGTTG
tph_qpcr         AACTCGCCACGCTCTACTGGTTCACTGTTG
tph_cloned       AACTCGCCACGCTCTACTGGTTCACTGTTG
                 AACTCGCCACGCTCTACTGGTTCACTGTTG

                       1000      1010      1020
tph_nematostella AGTTTGGGCTGTGTCGGCAGGAGGGCGAGG
tph_qpcr         AGTTTGGGCTGTGTCGGCAGGAGGGCGAGG
tph_cloned       AGTTTGGGCTGTGTCGGCAGGAGGGCGAGG
                 AGTTTGGGCTGTGTCGGCAGGAGGGCGAGG

                       1030      1040      1050
tph_nematostella TGAAAGCGTACGGCGCTGGTCTATTGTCCT
tph_qpcr         TGAAAGCGTACGGCGCTGGTCTATTGTCCT
tph_cloned       TGAAAGCGTACGGCGCTGGTCTATTGTCCT
                 TGAAAGCGTACGGCGCTGGTCTATTGTCCT

                       1060      1070      1080
tph_nematostella CCTTTGGGGAGTTACAGTATTGCTTGACCG
tph_qpcr         CCTTTGGGGAGTTACAGTATTG--------
tph_cloned       CCTTTGGGGAGTTACAGTATG-CTTGACCG
                 CCTTTGGGGAGTTACAGTATTGCTTGACCG

                       1090      1100      1110
tph_nematostella ATGAGCCCAAGAAATACCCTCTGGAGCCTG
tph_qpcr         ------------------------------
tph_cloned       ATGAGCCCAAGAAATACCCTCTGGAGCCTG
                 ATGAGCCCAAGAAATACCCTCTGGAGCCTG

                       1120      1130      1140
tph_nematostella AAAAGACCAGTGTACAGAAGTACCCCATCA
tph_qpcr         ------------------------------
tph_cloned       AAAAGACCAGTGTACAGAAGTACCCCATCA
                 AAAAGACCAGTGTACAGAAGTACCCCATCA

                       1150      1160      1170
tph_nematostella CGCAGTACCAGCCAGTCTACTTCATTGCCG
tph_qpcr         ------------------------------
tph_cloned       CGCAGTACCAGCCAGTCTACTTCATTGCCG
                 CGCAGTACCAGCCAGTCTACTTCATTGCCG

                       1180      1190      1200
tph_nematostella ACAGTTTCTTGTCGGCCAGAAACAAAGTTA
tph_qpcr         ------------------------------
tph_cloned       ACAGTTTCTTGTCGGCCAGAAACAAAGTTA
                 ACAGTTTCTTGTCGGCCAGAAACAAAGTTA

                       1210      1220      1230
tph_nematostella GGAAACCCTGTTTTATTACCAAGGAATAGA
tph_qpcr         ------------------------------
tph_cloned       GGAAACCCTGTTTTATTACCAAGGAATAGA
                 GGAAACCCTGTTTTATTACCAAGGAATAGA

                       1240      1250      1260
tph_nematostella ACTTAAGCTTATAAGCAATGTATTGCAAGG
tph_qpcr         ------------------------------
tph_cloned       ACTTAAGCTTATAAGCAATGTATTGCAAGG
                 ACTTAAGCTTATAAGCAATGTATTGCAAGG

                       1270      1280      1290
tph_nematostella CCTTGTAGTTTTATAACAGCAAGAATTGTA
tph_qpcr         ------------------------------
tph_cloned       CCTTGTAGTTTTATAACAGCAAGAATTGTA
                 CCTTGTAGTTTTATAACAGCAAGAATTGTA

                       1300      1310      1320
tph_nematostella GGAAGAATTTTGTGCAATATATGTAAAGGT
tph_qpcr         ------------------------------
tph_cloned       GGAAGAATTTTGTGCAATATATGTAAAGGT
                 GGAAGAATTTTGTGCAATATATGTAAAGGT

                       1330      1340      1350
tph_nematostella ATTCTTAGGCAAGGGAATATGTGTTAAGGA
tph_qpcr         ------------------------------
tph_cloned       ATTCTTAGGCAAGGGAATATGTGTTAAGGA
                 ATTCTTAGGCAAGGGAATATGTGTTAAGGA

                       1360      1370      1380
tph_nematostella CTGTTTTGTTGTGATTTTAGAAAACTATCC
tph_qpcr         ------------------------------
tph_cloned       CTGTTTTGTTGTGATTTTAGAAAACTATCC
                 CTGTTTTGTTGTGATTTTAGAAAACTATCC

                       1390      1400      1410
tph_nematostella CCCATCCCTTTCCCTCCATTGGTAATGTAG
tph_qpcr         ------------------------------
tph_cloned       CCCATCCCTTTCCCTCCATTGGTAATGTAG
                 CCCATCCCTTTCCCTCCATTGGTAATGTAG

                       1420      1430      1440
tph_nematostella TGATATCAAGAAAAAATTCATTAGGTATTT
tph_qpcr         ------------------------------
tph_cloned       TGATATCAAGAAAAAATTCATTAGGTATTT
                 TGATATCAAGAAAAAATTCATTAGGTATTT

                       1450      1460      1470
tph_nematostella AAGCTCTGTGTTATTTATAGACTGGATTTT
tph_qpcr         ------------------------------
tph_cloned       AAGCTCTGTGTTATTTATAGACTGGATTTT
                 AAGCTCTGTGTTATTTATAGACTGGATTTT

                       1480      1490      1500
tph_nematostella TCCCGACCTGTAGTATCTTAAAGACCGGTA
tph_qpcr         ------------------------------
tph_cloned       TCCCGACCTGTAGTATCTTAAAGACCGGTA
                 TCCCGACCTGTAGTATCTTAAAGACCGGTA

                       1510      1520      1530
tph_nematostella GTATCTTAAGATCTGTGGATTAAACTTTTT
tph_qpcr         ------------------------------
tph_cloned       GTATCTTAAGATCTGTGGATTAAACTTTTT
                 GTATCTTAAGATCTGTGGATTAAACTTTTT

                       1540      1550      1560
tph_nematostella ATTAAAAGAACCATTATCACCCACACCGTT
tph_qpcr         ------------------------------
tph_cloned       ATTAAAAGAACCATTATCACCCACACCGTT
                 ATTAAAAGAACCATTATCACCCACACCGTT

                       1570      1580      1590
tph_nematostella GTTATTGTTTCACAATATTAACCATAAACA
tph_qpcr         ------------------------------
tph_cloned       GTTATTGTTTCACAATATTAACCATAAACA
                 GTTATTGTTTCACAATATTAACCATAAACA

                       1600      1610      1620
tph_nematostella CAAGAACAATATCCACAGCTTACAGTGTCA
tph_qpcr         ------------------------------
tph_cloned       CAAGAACAATATCCACAGCTTACAGTGTCA
                 CAAGAACAATATCCACAGCTTACAGTGTCA

                       1630      1640      1650
tph_nematostella CAATGTTTTAAGGAGTGCACTGTCTGATTA
tph_qpcr         ------------------------------
tph_cloned       CAATGTTTTAAGGAGTGCACTGTCTGATTA
                 CAATGTTTTAAGGAGTGCACTGTCTGATTA

                       1660      1670      1680
tph_nematostella AAATGAACTTAAATAACTGGTGTGGCAAAG
tph_qpcr         ------------------------------
tph_cloned       AAATGAACTTAAATAACTGGTGTGGCAAAG
                 AAATGAACTTAAATAACTGGTGTGGCAAAG

                       1690      1700      1710
tph_nematostella GATGATTCAATGCACATCAGAACAGGTGTG
tph_qpcr         ------------------------------
tph_cloned       GATGATTCAATGCACATCAGAACAGGTGTG
                 GATGATTCAATGCACATCAGAACAGGTGTG

                       1720      1730      1740
tph_nematostella TGCCCGGGATTGGCTGATGGGGGTGCACAG
tph_qpcr         ------------------------------
tph_cloned       TGCCCGGGATTGGCTGATGGGGGTGCACAG
                 TGCCCGGGATTGGCTGATGGGGGTGCACAG

                       1750      1760      1770
tph_nematostella TCATAGGTATATGGAAAGAGCATTGTATTT
tph_qpcr         ------------------------------
tph_cloned       TCATAGGTATATGGAAAGAGCATTGTATTT
                 TCATAGGTATATGGAAAGAGCATTGTATTT

                       1780      1790      1800
tph_nematostella GAAGACCAACTTTTTTTTTTGGGGGGGGGG
tph_qpcr         ------------------------------
tph_cloned       GAAGACCAACTTTTTTTTTTGGGGGGGGGG
                 GAAGACCAACTTTTTTTTTTGGGGGGGGGG

                       1810      1820      1830
tph_nematostella GGGGGAGAGGGTGATCGTGCACCCTGTGCA
tph_qpcr         ------------------------------
tph_cloned       GGGGGAGAGGGTGATCGTGCACCCTGTGCA
                 GGGGGAGAGGGTGATCGTGCACCCTGTGCA

                       1840      1850      1860
tph_nematostella CCCCCCTGCGTCTGCACCTGCAGAATATGA
tph_qpcr         ------------------------------
tph_cloned       CCCCCCTGCGTCTGCACCTGCAGAATATGA
                 CCCCCCTGCGTCTGCACCTGCAGAATATGA

                       1870      1880      1890
tph_nematostella TGGACATTTCCAAGTTATCAACTTAATCCA
tph_qpcr         ------------------------------
tph_cloned       TGGACATTTCCAAGTTATCAACTTAATCCA
                 TGGACATTTCCAAGTTATCAACTTAATCCA

                       1900      1910      1920
tph_nematostella GTGATTCTTGCGACCTAATTAGCTAACTTC
tph_qpcr         ------------------------------
tph_cloned       GTGATTCTTGCGACCTAATTAGCTAACTTC
                 GTGATTCTTGCGACCTAATTAGCTAACTTC

                       1930      1940      1950
tph_nematostella CACTTTGACAATGGTGGCAACGGCATTATT
tph_qpcr         ------------------------------
tph_cloned       CACTTTGACAATGGTGGCAACGGCATTATT
                 CACTTTGACAATGGTGGCAACGGCATTATT

                       1960      1970      1980
tph_nematostella TGATAACCGTCTTATCATCTGAAATTGAAA
tph_qpcr         ------------------------------
tph_cloned       TGATAACCGTCTTATCATCTGAAATTGAAA
                 TGATAACCGTCTTATCATCTGAAATTGAAA

                       1990      2000      2010
tph_nematostella GAGCTGCTCATGAAACATTAAATTATATAT
tph_qpcr         ------------------------------
tph_cloned       GAGCTGCTCATGAAACATTAAATTATAT--
                 GAGCTGCTCATGAAACATTAAATTATATAT

                       2020      2030      2040
tph_nematostella GCCTATTTCAAAT
tph_qpcr         -------------
tph_cloned       -------------
                 GCCTATTTCAAAT
```
