# Supplementary material for: Developmental and light-entrained expression of melatonin and its relationship to the circadian clock in the sea anemone Nematostella vectensis
Source: EvoDevo. 2014 Aug 14;5:26. doi: 10.1186/2041-9139-5-26 (PMC4169136; doi:10.1186/2041-9139-5-26)
Supplement: Additional file 4 — Formatted Alignments of the sequences of HIOMT gene in Nematostella vectensis genome, our clone and the qPCR amplified fragment. [file 2041-9139-5-26-S4.pdf]

Formatted Alignments

```
                    10        20        30
hiomt_nematostella  CTTTCTCATTGTGAAATGGCAGAATTGACA
hiomt_qpcr          ------------------------------
hiomt_cloned        -----TCATTGTGAAATGGCAGAATTGACA
                    CTTTCTCATTGTGAAATGGCAGAATTGACA

                    40        50        60
hiomt_nematostella  AGTACTCTCGGTACAATCATACCTACAGCC
hiomt_qpcr          ------------------------------
hiomt_cloned        AGTACTCTCGGTACAATCATACCTACAGCC
                    AGTACTCTCGGTACAATCATACCTACAGCC

                    70        80        90
hiomt_nematostella  CTTTCAGATATTTTTCATGGATTTGTTAAA
hiomt_qpcr          ------------------------------
hiomt_cloned        CTTTCAGATATTTTTCATGGATTTGTTAAA
                    CTTTCAGATATTTTTCATGGATTTGTTAAA

                    100       110       120
hiomt_nematostella  TCAAAAGTATTGTTCACCGCCTGTGAATTA
hiomt_qpcr          ------------------------------
hiomt_cloned        TCAAAAGTATTGTTCACCGCCTGTGAATTA
                    TCAAAAGTATTGTTCACCGCCTGTGAATTA

                    130       140       150
hiomt_nematostella  GGTATATTTGACGTACTGTGCGATAAGCAT
hiomt_qpcr          ------------------------------
hiomt_cloned        GGTATATTTGACGTACTGTGCGATAAGCAT
                    GGTATATTTGACGTACTGTGCGATAAGCAT

                    160       170       180
hiomt_nematostella  TCGGCAGAACATGTGGCAGAAAATGTTAAA
hiomt_qpcr          ------------------------------
hiomt_cloned        TCGGCAGAACATGTGGCAGAAAATGTTAAA
                    TCGGCAGAACATGTGGCAGAAAATGTTAAA

                    190       200       210
hiomt_nematostella  CCAAGCTGCAAAACTGGCTCAACTCGTCGT
hiomt_qpcr          ------------------------------
hiomt_cloned        CCAAGCTGCAAAACTGGCTCAACTCGTCGT
                    CCAAGCTGCAAAACTGGCTCAACTCGTCGT

                    220       230       240
hiomt_nematostella  CTTCTTGACACACTTGTAGCTATGCAATTA
hiomt_qpcr          ------------------------------
hiomt_cloned        CTTCTTGACACACTTGTAGCTATGCAATTA
                    CTTCTTGACACACTTGTAGCTATGCAATTA

                    250       260       270
hiomt_nematostella  CTTGTCAAAGAAATGGACAGCGACCCGCCT
hiomt_qpcr          ------------------------------
hiomt_cloned        CTTGTCAAAGAAATGGACAGCGACCCGCCT
                    CTTGTCAAAGAAATGGACAGCGACCCGCCT

                    280       290       300
hiomt_nematostella  GTCTACTTCAATAGCCAAACTGCAGAGGCA
hiomt_qpcr          ------------------------------
hiomt_cloned        GTCTACTTCAATAGCCAAACTGCAGAGGCA
                    GTCTACTTCAATAGCCAAACTGCAGAGGCA

                    310       320       330
hiomt_nematostella  TTTCTTACTAGGAAAAGTCCCAAGTCTCTC
hiomt_qpcr          ------------------------------
hiomt_cloned        TTTCTTACTAGGAAAAGTCCCAAGTCTCTC
                    TTTCTTACTAGGAAAAGTCCCAAGTCTCTC

                    340       350       360
hiomt_nematostella  ATAAGCTACTTTGAATTTCTTCACGCCACC
hiomt_qpcr          ------------------------------
hiomt_cloned        ATAAGCTACTTTGAATTTCTTCACGCCACC
                    ATAAGCTACTTTGAATTTCTTCACGCCACC

                    370       380       390
hiomt_nematostella  GATTACAAGCTATTTGACAACCTGAAGCAT
hiomt_qpcr          ------------------------------
hiomt_cloned        GATTACAAGCTATTTGACAACCTGAA-CAT
                    GATTACAAGCTATTTGACAACCTGAAGCAT

                    400       410       420
hiomt_nematostella  GCGGTTGTCGAGGAGCAGCCACAATGGCAA
hiomt_qpcr          ------------------------------
hiomt_cloned        GCGGTTGTCGAGGAGCAGCCACAATGGCAA
                    GCGGTTGTCGAGGAGCAGCCACAATGGCAA

                    430       440       450
hiomt_nematostella  CGAGCCTTCGATCAGCCGGCTTCAGAAATT
hiomt_qpcr          ------------------------------
hiomt_cloned        CGAGCCTTCGATCAGCCGGCTTCAGAAATT
                    CGAGCCTTCGATCAGCCGGCTTCAGAAATT

                    460       470       480
hiomt_nematostella  TACAAAATAGTGCATTCCAATAAAGAAGAG
hiomt_qpcr          ------------------------------
hiomt_cloned        TACAAAATAGTGCATTCCAATAAAGAAGAG
                    TACAAAATAGTGCATTCCAATAAAGAAGAG

                    490       500       510
hiomt_nematostella  CTGGTGCGTATCTACTCGAGATTTGATTGC
hiomt_qpcr          ------------------AGATTTGATTGC
hiomt_cloned        CTGGTGCGTATCTACTCGAGATTTGATTGC
                    CTGGTGCGTATCTACTCGAGATTTGATTGC

                    520       530       540
hiomt_nematostella  GTTCACCGATTGGAAGCACCTGGGGTGATG
hiomt_qpcr          GTTCACCGATTGGAAGCACCTGGGGTGATG
hiomt_cloned        GTTCACCGATTGGAAGCACCTGGGGTGATG
                    GTTCACCGATTGGAAGCACCTGGGGTGATG

                    550       560       570
hiomt_nematostella  TCTGCGTTTGACTTGAACCCTTTTAAACAC
hiomt_qpcr          TCTGCGTTTGACTTGAACCCTTTTAAACAC
hiomt_cloned        TCTGCGTTTGACTTGAACCCTTTTAAACAC
                    TCTGCGTTTGACTTGAACCCTTTTAAACAC

                    580       590       600
hiomt_nematostella  ATGTGTGATCTTGGAGGAGGAACTGGCTGT
hiomt_qpcr          ATGTGTGATCTTGGAGGAGGAACTGGCTGT
hiomt_cloned        ATGTGTGATCTTGGAGGAGGAACTGGCTGT
                    ATGTGTGATCTTGGAGGAGGAACTGGCTGT

                    610       620       630
hiomt_nematostella  TTTTCATACGAGGCGTGCAAGCAGTATCCT
hiomt_qpcr          T-----------------------------
hiomt_cloned        TTTTCATACGAGGCGTGCAAGCAGTATCCT
                    TTTTCATACGAGGCGTGCAAGCAGTATCCT

                    640       650       660
hiomt_nematostella  GCATTAAAAATCACAATTTACGAAATGCAG
hiomt_qpcr          ------------------------------
hiomt_cloned        GCATTAAAAATCACAATTTACGAAATGCAG
                    GCATTAAAAATCACAATTTACGAAATGCAG

                    670       680       690
hiomt_nematostella  CCAATCTTAGATGTCGCGCCTGCTTTCAAG
hiomt_qpcr          ------------------------------
hiomt_cloned        CCAATCTTAGATGTCGCGCCTGCTTTCAAG
                    CCAATCTTAGATGTCGCGCCTGCTTTCAAG

                    700       710       720
hiomt_nematostella  CCAACCATCGCTGATTGCCCTAACCAATCA
hiomt_qpcr          ------------------------------
hiomt_cloned        CCAACCATCGCTGATTGCCCTAACCAATCA
                    CCAACCATCGCTGATTGCCCTAACCAATCA

                    730       740       750
hiomt_nematostella  AACGTCACCTACGTAGCGGGAGACTTTTTT
hiomt_qpcr          ------------------------------
hiomt_cloned        AACGTCACCTACGTAGCGGGAGACTTTTTT
                    AACGTCACCTACGTAGCGGGAGACTTTTTT

                    760       770       780
hiomt_nematostella  AAAGACCCACTTCCGGTTGCTGACCTGTAT
hiomt_qpcr          ------------------------------
hiomt_cloned        AAAGACCCACTTCCGGTTGCTGACCT-TAT
                    AAAGACCCACTTCCGGTTGCTGACCTGTAT

                    790       800       810
hiomt_nematostella  TTTCTGGCCCATGTTCTCCACAATTGGGCA
hiomt_qpcr          ------------------------------
hiomt_cloned        TTTCTGGCCCATGTTCTCCACAATTGGGCA
                    TTTCTGGCCCATGTTCTCCACAATTGGGCA

                    820       830       840
hiomt_nematostella  GAAGAAAAGGTTGACTTGCTACTTAGCAAA
hiomt_qpcr          ------------------------------
hiomt_cloned        GAAGAAAAGGTTGACTTGCTACTTAGCAAA
                    GAAGAAAAGGTTGACTTGCTACTTAGCAAA

                    850       860       870
hiomt_nematostella  GTTTTTGCAGTGCTGCCACCAGGTGGAGGT
hiomt_qpcr          ------------------------------
hiomt_cloned        GTTTTTGCAGTGCTGCCACCAGGTGGAGGT
                    GTTTTTGCAGTGCTGCCACCAGGTGGAGGT

                    880       890       900
hiomt_nematostella  ATACTTCTTGGGGAAGTTCTTCTTCCTGTT
hiomt_qpcr          ------------------------------
hiomt_cloned        ATACTTCTTGGGGAAGTTCTTCTTCCTGTT
                    ATACTTCTTGGGGAAGTTCTTCTTCCTGTT

                    910       920       930
hiomt_nematostella  GACGAGCCGAATCCACAGCTCTCTGCACCC
hiomt_qpcr          ------------------------------
hiomt_cloned        GACGAGCCGAATCCACAGCTCTCTGCACCC
                    GACGAGCCGAATCCACAGCTCTCTGCACCC

                    940       950       960
hiomt_nematostella  TTCCTGGACCTCACCATGCTAGTGACCTGC
hiomt_qpcr          ------------------------------
hiomt_cloned        TTCCTGGACCTCACCATGCTAGTGACCTGC
                    TTCCTGGACCTCACCATGCTAGTGACCTGC

                    970       980       990
hiomt_nematostella  GAGTCAGGGGCACGTGACCGTTCGGGCCCC
hiomt_qpcr          ------------------------------
hiomt_cloned        GAGTCAGGGGCACGTGACCGTTCGGGCCCC
                    GAGTCAGGGGCACGTGACCGTTCGGGCCCC

                    1000      1010      1020
hiomt_nematostella  GAGTACAAACGATTACTGGAAAGACATGGA
hiomt_qpcr          ------------------------------
hiomt_cloned        GAGTACAAACGATTACTGGAAAGACATGGA
                    GAGTACAAACGATTACTGGAAAGACATGGA

                    1030      1040      1050
hiomt_nematostella  TTTCAAGACGTGCGGATGAAATCCTTGCCA
hiomt_qpcr          ------------------------------
hiomt_cloned        TTTCAAGACGTGCGGATGAAATCCTTGCCA
                    TTTCAAGACGTGCGGATGAAATCCTTGCCA

                    1060      1070      1080
hiomt_nematostella  GGCGCCAAAACGACCGATGCTGTTTTCGCC
hiomt_qpcr          ------------------------------
hiomt_cloned        GGCGCCAAAACGACCGATGCTGTTTTCGCC
                    GGCGCCAAAACGACCGATGCTGTTTTCGCC

                    1090      1100      1110
hiomt_nematostella  CGGAAACCTTAAAATCGCCACGGCGCGACG
hiomt_qpcr          ------------------------------
hiomt_cloned        CGGAAACCTTAAAATCGCCACGGCGCGACG
                    CGGAAACCTTAAAATCGCCACGGCGCGACG

                    1120      1130      1140
hiomt_nematostella  GCACTTATACTTTACCATACGTAATTTTCT
hiomt_qpcr          ------------------------------
hiomt_cloned        GCACTTATACTTTACCATACGTAATTTTCT
                    GCACTTATACTTTACCATACGTAATTTTCT

                    1150      1160      1170
hiomt_nematostella  ACGACAGCTTGTGAACAAAATTACAAAATA
hiomt_qpcr          ------------------------------
hiomt_cloned        ACGACAGCTTGTGAACAAAATTACAAAATA
                    ACGACAGCTTGTGAACAAAATTACAAAATA

                    1180      1190      1200
hiomt_nematostella  TCCGCTTTGGCATTAAACC
hiomt_qpcr          -------------------
hiomt_cloned        TCCGCTTTG----------
                    TCCGCTTTGGCATTAAACC
```
