# Supplementary figures and images for: Developmental and light-entrained expression of melatonin and its relationship to the circadian clock in the sea anemone Nematostella vectensis
Source: EvoDevo. 2014 Aug 14;5:26. doi: 10.1186/2041-9139-5-26 (PMC4169136; doi:10.1186/2041-9139-5-26)

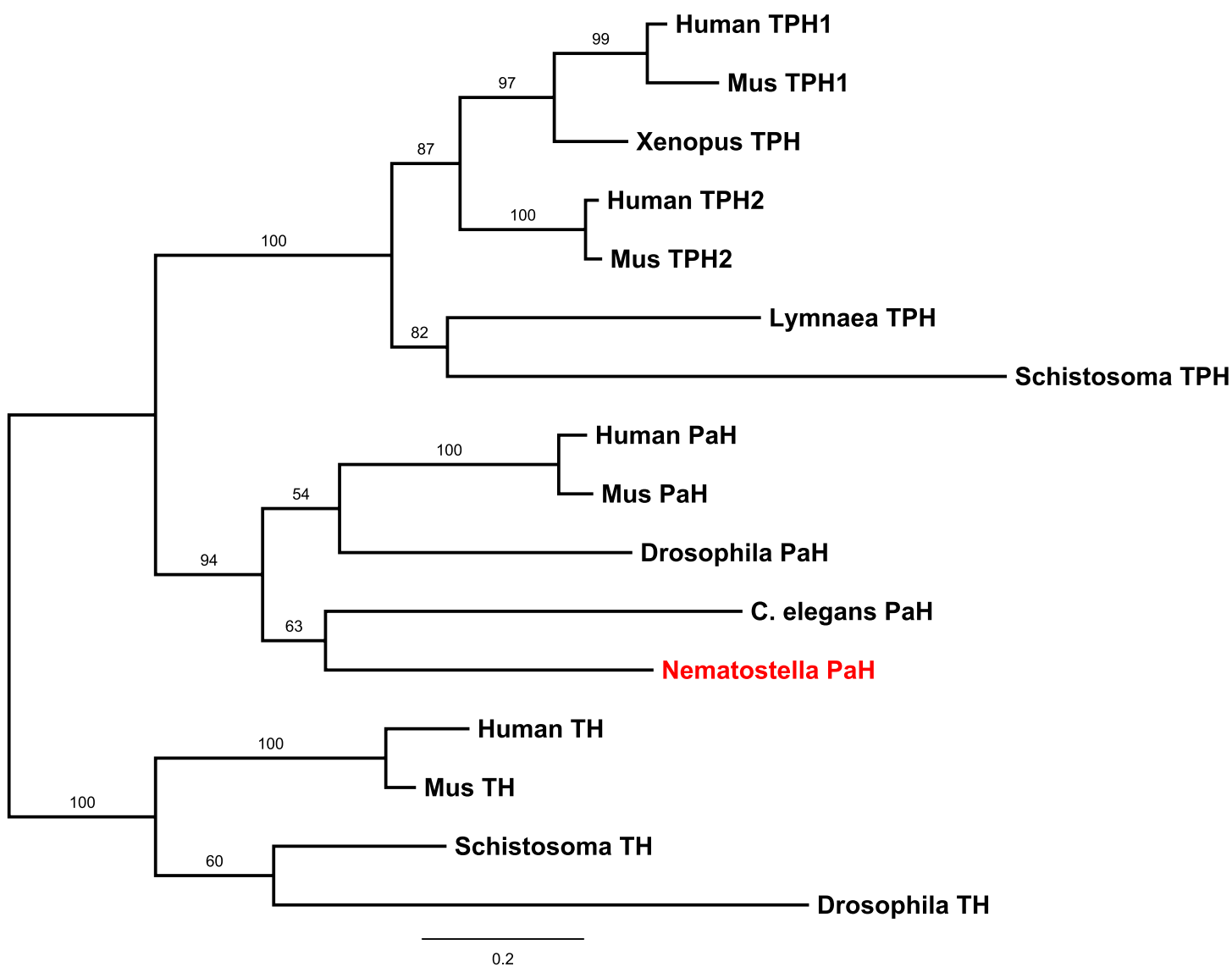

Supplement: Additional file 5 — Phylogenetic analyses of TPH gene. [file 2041-9139-5-26-S5.pdf]

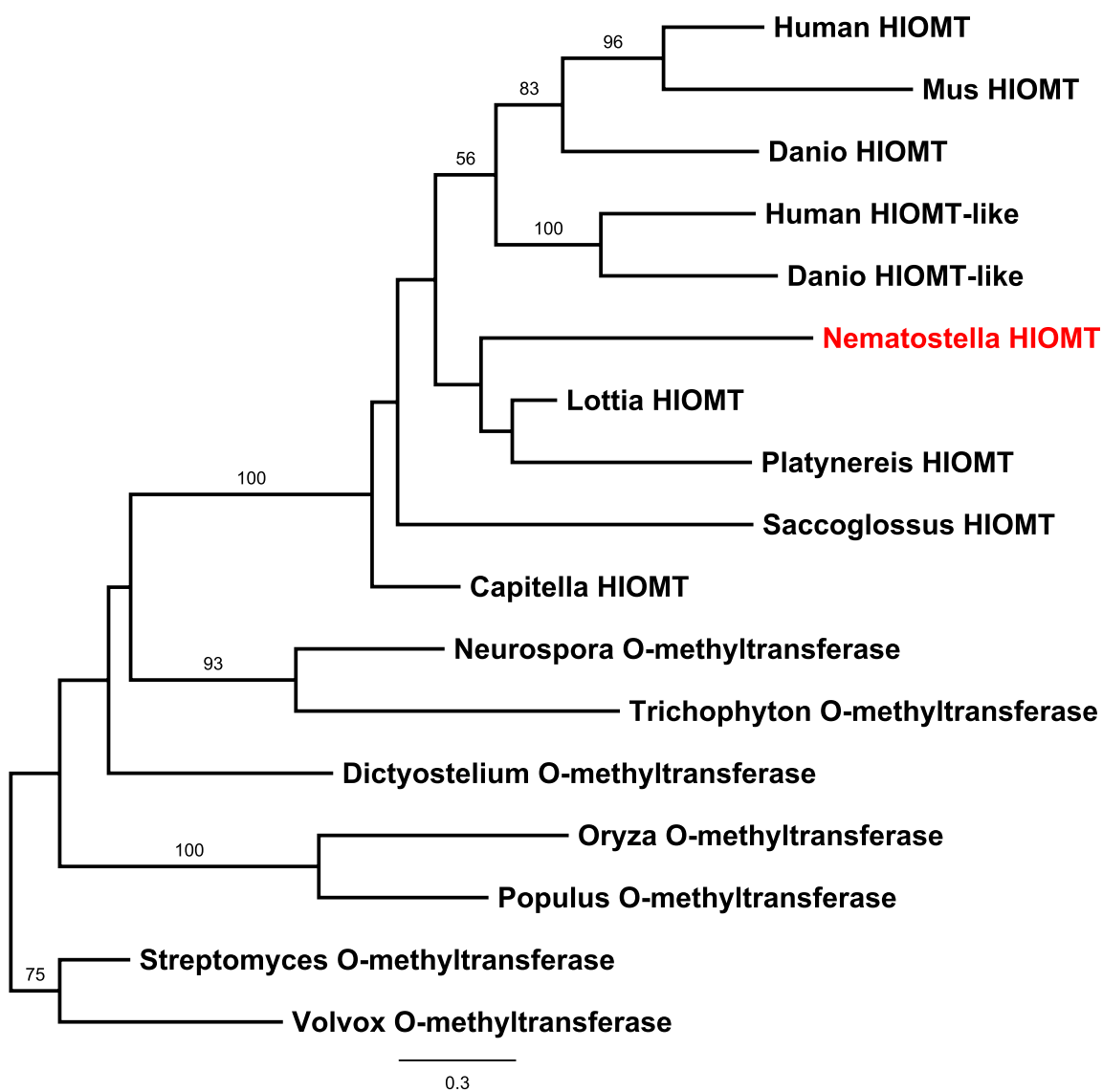

Supplement: Additional file 6 — Phylogenetic analyses of HIOMT gene. [file 2041-9139-5-26-S6.pdf]
